# Highly Efficient Delivery of Novel MiR-13896 by Human Umbilical Cord Mesenchymal Stem Cell-Derived Small Extracellular Vesicles Inhibits Gastric Cancer Progression by Targeting ATG2A-Mediated Autophagy

**DOI:** 10.34133/bmr.0119

**Published:** 2024-12-18

**Authors:** Peipei Wu, Min Wang, Can Jin, Linli Li, Yuting Tang, Zhangfei Wang, Xianwen Wang, Wenrong Xu, Hui Qian

**Affiliations:** ^1^ Department of Laboratory Medicine, The First Affiliated Hospital of USTC, Division of Life Sciences and Medicine, University of Science and Technology of China, Hefei, Anhui 230001, China.; ^2^ Core Unit of National Clinical Research Center for Laboratory Medicine, Hefei, Anhui 230001, China.; ^3^ School of Biomedical Engineering, Research and Engineering Center of Biomedical Materials, Anhui Medical University, Hefei, Anhui 230032, China.; ^4^ Jiangsu Key Laboratory of Medical Science and Laboratory Medicine, Department of Clinical Laboratory, School of Medicine, Jiangsu University, Zhenjiang, Jiangsu 212000, China.; ^5^ Department of Clinical Laboratory, Changzhou Second Hospital, Changzhou, Jiangsu 213000, China.

## Abstract

Gastric cancer (GC) is the fourth most common cancer and the second leading cause of cancer-related deaths worldwide. Despite recent advancements, clinical outcomes for GC remain unsatisfactory. Mesenchymal stem cell (MSC)-derived extracellular vesicles (EVs) have shown promise in inhibiting tumor progression, but their role in GC, specifically human umbilical cord MSC-derived small EVs (hucMSC-sEVs), is not well understood. This study investigates the therapeutic potential of hucMSC-sEVs in GC treatment. We found that hucMSC-sEVs are captured by GC cells, substantially inhibiting their proliferation and inducing apoptosis. MiRNA sequencing revealed that hucMSC-sEVs were enriched with miRNAs having anticancer properties. Among these, miR-13896, a new miRNA, was identified as a potent inhibitor of GC cell proliferation and a promoter of apoptosis. Mechanistic studies revealed that miR-13896 targets and down-regulates the ATG2A-mediated autophagy pathway, suppressing GC cell growth and metastasis. Furthermore, we enriched hucMSC-sEVs with miR-13896 through electroporation. These engineered EVs specifically targeted tumor sites and significantly reduced GC cell growth and migration in vitro and in vivo. MiR-13896 emerged as a promising therapeutic target for GC. The delivery of miR-13896 via hucMSC-sEVs represents a novel and effective strategy for GC treatment, highlighting the potential of EV-based therapies to combat this malignancy.

## Introduction

Gastric cancer (GC) is the fifth most prevalent malignancy worldwide and the third leading cause of cancer-related mortality [1,2]. Due to its insidious onset, elusive early symptoms, rapid progression, and high mortality rate, GC has become a significant public health concern and a socioeconomic burden. Despite substantial advances in therapeutic strategies over recent decades, including surgery, chemotherapy, radiotherapy, hyperthermia, and biological therapy, clinical outcomes for patients with GC remain unsatisfactory. These treatments often result in postoperative recurrence and severe side effects, contributing to a 5-year survival rate of less than 40% [3]. Therefore, there is a critical need for new therapeutic targets and effective strategies to delay the malignant progression of GC.

Mesenchymal stem cells (MSCs) have attracted considerable attention in cancer research due to their dual role in promoting and inhibiting tumor growth. Although the tumor-modulatory effects of adipose and bone marrow-derived MSCs remain controversial, human umbilical cord MSCs (hucMSCs) have consistently demonstrated the ability to suppress tumor growth both directly and indirectly. This distinction underscores the therapeutic potential of hucMSCs, particularly in the production of extracellular vesicles (EVs) that can be used for cancer treatment. EVs are nano-to-micrometer-scale membrane-bound vesicles secreted by cells that encapsulate bioactive molecules such as proteins and nucleic acids. Among these, small EVs (sEVs), which include exosomes, have garnered significant research interest due to their roles in cell communication and tumor inhibition [4]. In recent decades, MSC-EVs, including sEVs, not only have shown strong therapeutic effects in tissue regeneration and repair but also have been gradually found to be effective in the intervention of complex and refractory tumor diseases. sEVs are a subgroup of EVs with a diameter of 30 to 200 nm; exosomes are the most abundant subtype of sEVs. Neonatal umbilical cord MSC-sEVs are natural lipid nanobilayer components with low immunogenicity and high biocompatibility, which is an excellent choice for drug delivery. In addition, some studies have confirmed that some active substances carried by unmodified MSC-sEVs have important anticancer effects. In particular, the potential of engineered sEVs in tumor-targeted therapy and drug delivery is constantly being explored by scientists. MSC-derived sEVs play a beneficial role in the precise and targeted therapy of various malignant tumors [5,6]. Proteins and nucleic acids rich in hucMSC-sEVs can be delivered to tumor cells and effectively inhibit the malignant progression of a variety of tumors. Among them, hucMSC-sEVs have unique advantages such as lower immunogenicity, enhanced safety, and efficient delivery of therapeutic agents [7]. In recent years, natural endogenous nanomaterials, sEVs, have been constructed using physical, chemical, and biological modification methods to enhance tumor-specific targeting of sEVs or to load them with potent therapeutic molecules, thus increasing their antitumor efficacy [8,9]. This engineering potential has broadened the application of sEVs in cancer therapy, offering new avenues for precision medicine.

Autophagy, a crucial cell process for maintaining homeostasis, involves the degradation and recycling of cellular components. ATG2A (autophagy-related protein homolog A) is a key regulator within this machinery and is essential for the formation and expansion of autophagosomes. The absence of ATG2A disrupts the completion of autophagosomes, leading to accumulation of immature autophagosomes and triggering atypical apoptosis pathways [10]. ATG2A can interact with different proteins to regulate autophagy and plays a key role in the development and occurrence of tumors such as liver cancer and brain glioma [11,12]. Xiao et al. [13] found that E2F transcription factor 4 (E2F4) facilitates cytoprotective autophagy via ATG2A in GC cells. Therefore, targeting ATG2A presents a promising strategy for precision GC therapy.

**Fig. 1.**
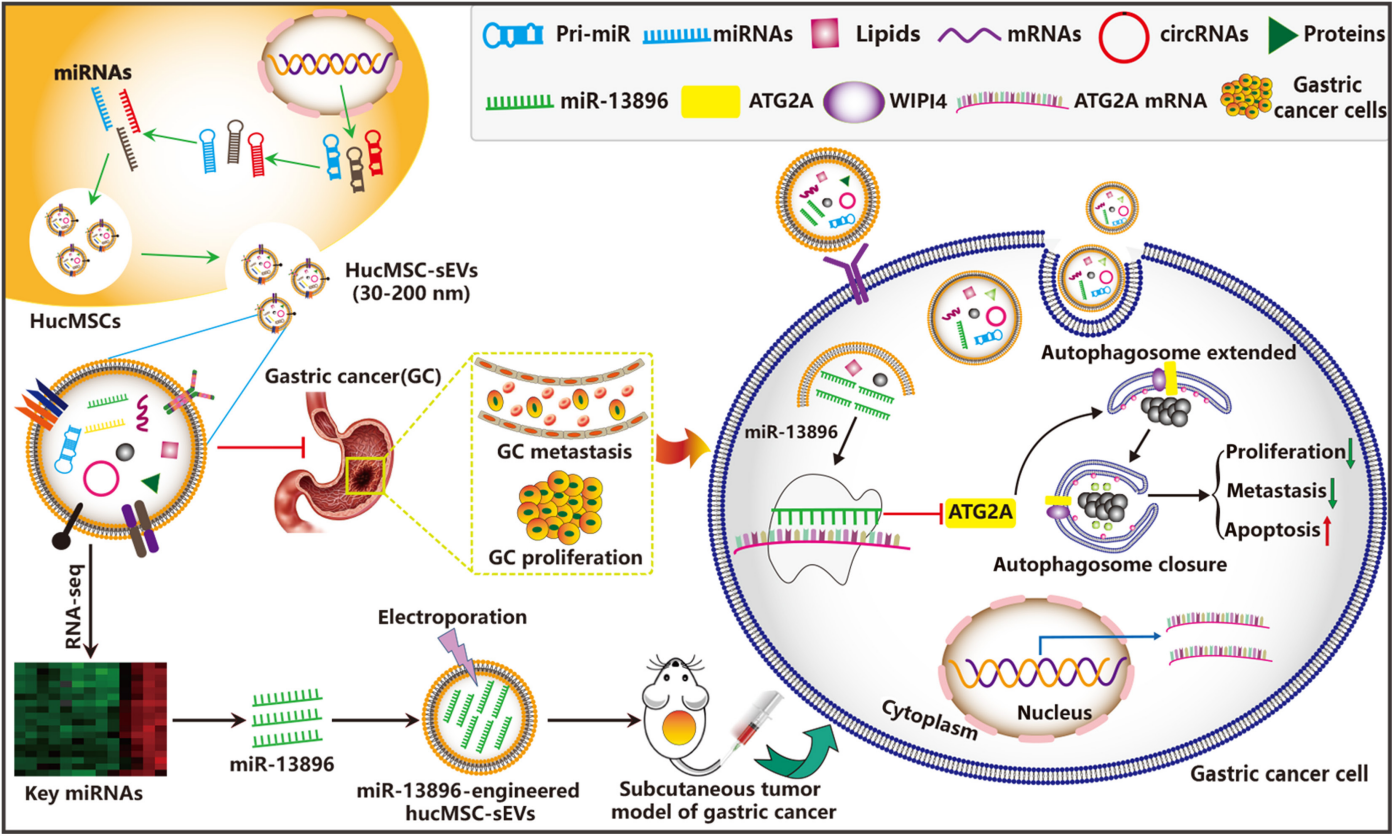
Schematic diagram for highly efficient delivery of novel miR-13896 by HucMSC-sEVs inhibits GC progression by targeting ATG2A-mediated autophagy.

Nucleotide components (including mRNA, miRNA, and circular RNA) enriched in MSC-sEVs are responsible for the treatment of many diseases. Through miRNA sequencing, the researchers found that MSC-derived EVs carry abundant miRNAs, including many new and undiscovered miRNAs. In addition, MSC-derived EV miRNAs also have also been found to inhibit the occurrence and progression of a variety of tumors. In this study, we investigated the therapeutic potential of hucMSC-sEVs in the context of GC. We reasoned that that hucMSC-sEVs can inhibit the proliferation and migration of GC cells and promote apoptosis in vitro. In addition, we found that autophagy plays a key role in hucMSC-sEV-mediated GC suppression. Mechanistically, we found that hucMSC-sEV shuttling a new miRNA-13896 (miR-13896) plays a significant role in the suppression of GC by targeting ATG2A and inhibiting autophagy. Therefore, this research highlights the critical role of ATG2A in GC progression and offers a novel approach to cancer therapy through the targeted delivery of miR-13896, paving the way for innovative treatments in oncology. Our findings suggest that miR-13896-loaded hucMSC-sEVs represent a promising therapeutic strategy for GC (Fig. 1).

## Materials and Methods

### Cell culture

After obtaining informed consent from the puerpera, umbilical cord tissue was collected from a newborn fetus from the Fourth People’s Hospital of Zhenjiang City. Primary hucMSCs were isolated as previously described [14]. Briefly, fresh umbilical cord tissues were immediately processed into 1-mm^3^-sized tissue blocks and affixed to sterile plates supplemented with minimal essential medium α (MEM-α) containing 10% fetal bovine serum (FBS) (Gibco, Grand Island, USA) at 37 °C with 5% CO_2_. After approximately 14 d, the primary cells were digested with 0.25% trypsin and then subcultured. The passaged cells were maintained with MEM-α containing 10% FBS and penicillin–streptomycin, and hucMSCs of generation P3 were used for subsequent experimental studies. The human mucosa epithelial cell line GES-1 was purchased from Gefan Biological Technology (Shanghai, China). Human GC cell lines MKN-45, HGC-27, SUN-1, and AGS were purchased from Runyan Biotechnology Company (Nanjing, China). Human embryonic kidney 293T cells (HEK-293T) were obtained from the Cell Bank of the Chinese Academy of Sciences (Shanghai, China). GES-1, MKN-45, and HGC-27 cells were cultured in RPMI 1640 (Bioind, Israel) with 10% FBS (Bovogen, Australia) at 37 °C with 5% CO_2_. AGS cells were cultured in Dulbecco’s modified Eagle’s medium/F-12 (Bioind, Israel) with 10% FBS (Bovogen, Australia) at 37 °C with 5% CO_2_.

### Western blotting

Protein extraction from cells and sEVs was performed using radioimmunoprecipitation assay buffer supplemented with protease inhibitors (Pierce, USA). Protein concentrations were quantified using a bicinchoninic acid (BCA) protein assay kit. Equal amounts of protein were subjected to 12% sodium dodecyl sulfate–polyacrylamide gel electrophoresis, transferred to polyvinylidene difluoride membranes (Millipore, USA), and blocked with 5% nonfat milk. Subsequently, the membranes were incubated with primary antibodies at 4 °C overnight. The membranes were washed with 1× tris-buffered saline with Tween (TBST) and then incubated with a horseradish peroxidase (HRP)-conjugated goat anti-rabbit/mouse immunoglobulin G (IgG) secondary antibody (Invitrogen, USA, 31460/31430) for 1 h at room temperature. Following washes, membranes were incubated with HRP-conjugated secondary antibodies (Invitrogen, USA) for 1 h at room temperature, and bands were visualized. The primary antibodies used in this study were as follows: Bax [Cell Signaling Technology (CST), USA, 2772S], Bcl2 (CST, USA, 15071S), PCNA (CST, USA, 4711S), CyclinD3 (CST, USA, 2936S), vimentin (CST, USA, 5741S), Snail (CST, USA, 3895S), Slug (CST, USA, 9585P), E-cadherin (CST, USA, 3195S), N-cadherin (CST, USA, 13116P), p-AKT (CST, USA, 4060S), t-AKT (SAB, USA, 21501), ATG2A (Invitrogen, USA, MA5-31639), LC3B (CST, USA, 2775S), Beclin1 (CST, USA, 3495S), P62 (CST, USA, 39749S), CD63 (Abcam, USA, ab59479), CD81 (Proteintech, USA, 18250-1-AP), Alix (CST, USA, 2171S), TSG101 (Abcam, USA, ab30871), calnexin (CST, USA, 4872S), and β-actin (Bioworld, USA, AP0060).

### RNA extraction and quantitative real-time polymerase chain reaction

Total RNA was extracted from cancer cells and tissues using Trizol reagent (Invitrogen, USA). cDNA was synthesized from 2 μg of RNA following the manufacturer’s protocol (Vazyme, China). Quantitative real-time polymerase chain reaction (qRT-PCR) was conducted on a QuantStudio 3 system (ABI, USA) using the SYBR Green PCR kit (CWBIO, China). Gene expression levels were calculated using the 2^−ΔΔCt^ method and normalized to β-actin, and primer sequences are provided in Table S1.

### Cellular uptake analysis

A 1-ml volume of hucMSC-sEVs was incubated with 5 μl of the membrane dye DIL (Thermo Fisher Scientific, USA, D3911) for 30 min at 37 °C. The stained hucMSC-sEVs were then transferred to 100-kDa MWCO (molecular weight cutoff) ultrafiltration centrifugal tubes (Millipore, USA). To remove unbound DIL, the samples were washed 3 times with phosphate-buffered saline (PBS) and centrifuged at 1,500*g* for 20 min. DIL-labeled hucMSC-sEVs with the same particle number were administered to the MKN-45 and HGC-27 cells. Laser scanning confocal microscopy was applied to identify internalization of the DIL-labeled hucMSC-sEVs by MKN-45 and HGC-27 cells at different time points.

### Isolation and morphological identification of hucMSC-sEVs

HucMSC-sEVs were isolated and purified as previously described [15]. The protein content of hucMSC-sEVs was determined using a BCA protein assay kit (Vazyme, China). The morphology of hucMSC-sEVs was observed by transmission electron microscopy (TEM; H-7800, Hitachi, Japan). Briefly, after mixing, 20 μl of hucMSC-sEVs was captured by a pipette and added to a special test paper to form a round droplet. A copper mesh with a diameter of 2 mm was placed upside down on the droplet surface. After standing at room temperature for 5 min, the excess liquid on the edge of the copper mesh was carefully removed with filter paper. The copper mesh was then placed upside down on a droplet of 30 g/l phosphotungstic acid (pH 6.8) and negatively stained for 5 min at room temperature. Finally, the copper mesh was dried under an incandescent lamp and placed under TEM for observation and photographing. Freshly extracted, unfrozen, and unthawed hucMSC-sEVs showed a “cup-shaped” structure under TEM. The morphology and height of hucMSC-sEVs were observed using an atomic force microscope (AFM; Bruker, Multimode VIII SPM, Germany). Briefly, after mixing using a pipette, 10 μl of freshly extracted hucMSC-sEVs was diluted with deionized water in an appropriate proportion. After the diluted hucMSC-sEVs were fully mixed, they were dripped onto the clean cell slides and dried overnight at room temperature. The next day, deionized water was added to the center of the slides 4 to 5 times to remove interference from the crystallization of the PBS salt. TEM revealed characteristic “cup-shaped” structures, while AFM provided detailed morphological and dimensional analysis.

### Colony formation assay

GC cells were harvested and seeded in 6-well plates and transfected with different treatment reagents for 48 h. A total of 2.5 × 10^3^ MKN-45 cells were seeded in 3.5-cm^2^ cell culture plates, and the complete fresh cell medium was replenished every 3 d. After approximately 14 d of culture, cells were washed with PBS 3 times and then 4% paraformaldehyde was added to fix the cells for 30 min at room temperature. After washing with PBS 3 times, crystal violet was added to stain the cells for 10 min at room temperature. The unbound dye was washed with PBS, the cells were dried, and the size and number of cell colonies were observed.

### Cell proliferation assay

GC cells (5 × 10^3^ cells per well) were seeded in 96-well plates. After cells had adhered for 12 to 24 h, different concentrations of hucMSC-sEVs or miR-13896 mimic/inhibitor were added to each well for processing at different times. The Cell Counting Kit-8 (CCK-8) assay was performed to evaluate cell proliferation activity according to the manufacturer’s procedures. A volume of 100 μl of medium containing 10% CCK-8 reagent (Vazyme, Nanjing, China) was added to each well and incubated in the dark for 2 h at 37 °C. The absorbance values of each well at 450 nm were measured by the Cytation 5 automatic microplate reader (BioTek, USA).

### Dual-luciferase reporter assay

MiR-13896 and the 3′ untranslated region binding site of ATG2A were predicted by mirBase. The luciferase reporter vector containing wild type (WT) or mutant type (MUT) of ATG2A was purchased from GenePharma (Suzhou, China). The MKN-45 and HEK-293T cells were cotransfected with miR-13896 mimic and 3 different luciferase reporter vectors for 24 h. Luciferase activity was detected using the Dual Luciferase Assay Kit (Promega, MA, USA) according to the manufacturer’s instructions. This experiment was performed at least 3 times.

### siRNA transfection

Small interference RNAs (siRNAs) from ATG2A and their negative controls (NCs) were obtained from GenePharma (Suzhou, China). MKN-45 and HGC-27 cells were seeded in a 6-well plate (2 × 10^5^ cells per well). After 24 h, siRNAs and their NCs were transfected into cells using Lipofectamine 2000 (Life Technologies, Shanghai, China) in serum-free medium. The cultured cells were switched to complete medium at 4 to 6 h after transfection and cultured for another 48 h. The siRNA sequences used are listed in Table S2.

### Hematoxylin and eosin staining, immunohistochemistry, and immunofluorescence staining

Tumor tissues and the heart, liver, spleen, lung, and kidney were obtained from euthanized mice. The samples were fixed in 4% paraformaldehyde and gradually dehydrated, embedded in paraffin, and then cut into 4-μm sections. The slides were dewaxed to water and prepared for hematoxylin and eosin (H&E) staining according to standard protocols. GC patient tissues and paired adjacent tissues (5 cm away from the tumor margin) were collected from Jiangbin Hospital Affiliated to Jiangsu University. Immunohistochemistry (IHC) staining was used to detect ATG2A (1:50, Invitrogen, USA, MA5-31639) in GC tissue, and IHC was performed according to the manufacturer’s instructions (Boster, USA, SA1020). Immunofluorescence (IF) was used to detect ATG2A (1:50, Invitrogen, USA, MA5-31639) and LC3B (1:50, USA, 2775S) expression. The secondary antibodies were Alexa Fluor 488-labeled anti-rabbit IgG (1:800, Invitrogen, USA) and Cy3-labeled anti-mouse IgG (1:800, CWBIO, Beijing, China). The stained slides were sealed and dried for observation. Images were acquired sequentially by microscopy (Nikon, Tokyo, Japan).

### Transwell migration assay

GC cells (1 × 10^5^ per well) were seeded in the upper chamber, and 10% FBS-containing medium was placed in the lower chamber. After incubation at 37 °C in 5% CO_2_ for 12 h, the cells remaining on the upper surface of the membrane were removed with a cotton swab. Cells that migrated through the 8-mm pores and adhered to the lower surface of the membrane were fixed with 4% paraformaldehyde, stained with crystal violet, and photographed.

### Flow cytometry for the detection of cell apoptosis

GC cells were collected from different treatment groups. The cells were washed with precooled PBS twice and then resuspended in 1 × 10^7^ cells/ml in 1× cell staining binding buffer; 100-μl cell suspension was added to the flow tube, and 5 μl of fluorescein isothiocyanate Annexin V was added followed by 10 μl of propidium iodide. The cells were mixed in a gentle vortex and then incubated at room temperature for 15 min away from light. Finally, 400 μl of Annexin V binding buffer was added to the flow tube and apoptosis was analyzed by flow cytometry after mixing.

### Preparation of hucMSC-sEVs-loaded miR-13896

Thirty-three micrograms of Cy5-labeled miR-13896 mimic NC and mimic powder was dissolved in a certain volume of diethyl pyrocarbonate (DEPC) water on a super-clean table and centrifuged at 3,000 rpm at room temperature for 5 min. The final concentration was then adjusted with DEPC water to a working concentration of 0.5 μg/μl. HucMSC-sEVs were diluted to a working concentration of 30 μg/μl with PBS, and the dissolved Cy5-labeled miR-13896 mimic NC and mimic were thoroughly combined with hucMSC-sEVs in a ratio of 1:9. For electroporation, the sample gun accurately absorbs the mixed solution of 50-μl mixed solution, and carefully added the solution along the side wall of the electrode cup, paying attention to avoid bubbles throughout. The electrode cup was placed in the electrode slot, and the power transfer conditions were set according to the following parameters: attenuation mode, perforation voltage 110 V, lasting 3 ms, resting 10 ms; drive voltage 25 V, continuous 50 ms, resting 50 ms; drive cycle number 10, capacitance 940 μF. The resistance of the solution to the appropriate range was adjusted, and electroporation was performed. After sample transfer, miR-13896 mimic NC and mimic-loaded hucMSC-sEVs were transferred to a new sterile 1.5-ml eppendorf (EP) tube. Subsequently, to restore the integrity of the lipid bilayer membrane of hucMSC-sEVs, samples were incubated in a cell incubator at 37 °C for 1 h.

### Construction and intervention of the GC bearing model

Twenty 4-week-old male nude mice with specific pathogen-free grade were purchased from Suzhou Cavens and commissioned to the Animal Center of Jiangsu University for the construction of gastric carcinoma subcutaneous tumor model. Each mouse was injected subcutaneously with 200 μl of PBS containing 1 × 10^7^ HGC-27 cells. All successful tumor-bearing mice were randomly divided into different treatment groups and given the corresponding treatment according to different animal experiments. The dose of each mouse was 1 × 10^10^ particles every 3 d. Tumor volume and weight were monitored every 3 d. Tumor size was measured with vernier calipers, and tumor volume was calculated as follows: tumor volume (mm^3^) = [length (mm) × width 2 (mm)]/2. Mice were euthanized (inhaled carbon monoxide) 20 d after treatment intervention, and tumor weight was measured by animal biopsy.

### Small-animal in vivo imaging

A 1-ml volume of hucMSC-sEVs was incubated with 5 μl of the membrane dye DIL (Thermo Fisher Scientific, USA, D3911) for 30 min at 37 °C. The labeled suspension of hucMSC-sEVs was transferred to a 100-kDa MWCO ultrafiltration centrifugal tube (Millipore, USA). To remove the unconjugated DIL, the samples were washed with PBS 3 times and centrifuged at 1,500*g* for 30 min. DIL-labeled hucMSC-sEVs were administered to tumor-bearing mice by tail vein, and small-animal in vivo imaging (PerkinElmer, USA) was used to detect the tumor tissue distribution of transplanted hucMSC-sEVs at 24 h.

### Statistical analysis

All data are presented as mean ± SD. Statistical analyses were conducted using GraphPad Prism software (version.9.5.1). Differences between 2 groups were evaluated using the Student’s *t* test with 2-tailed unpaired Student’s *t*, while comparisons between multiple groups were performed using one-way analysis of variance (ANOVA). A *P* value of <0.05 was considered statistically significant.

## Results and Discussion

### HucMSC-sEVs inhibited GC cell proliferation and promoted their apoptosis in vitro

**Fig. 2.**
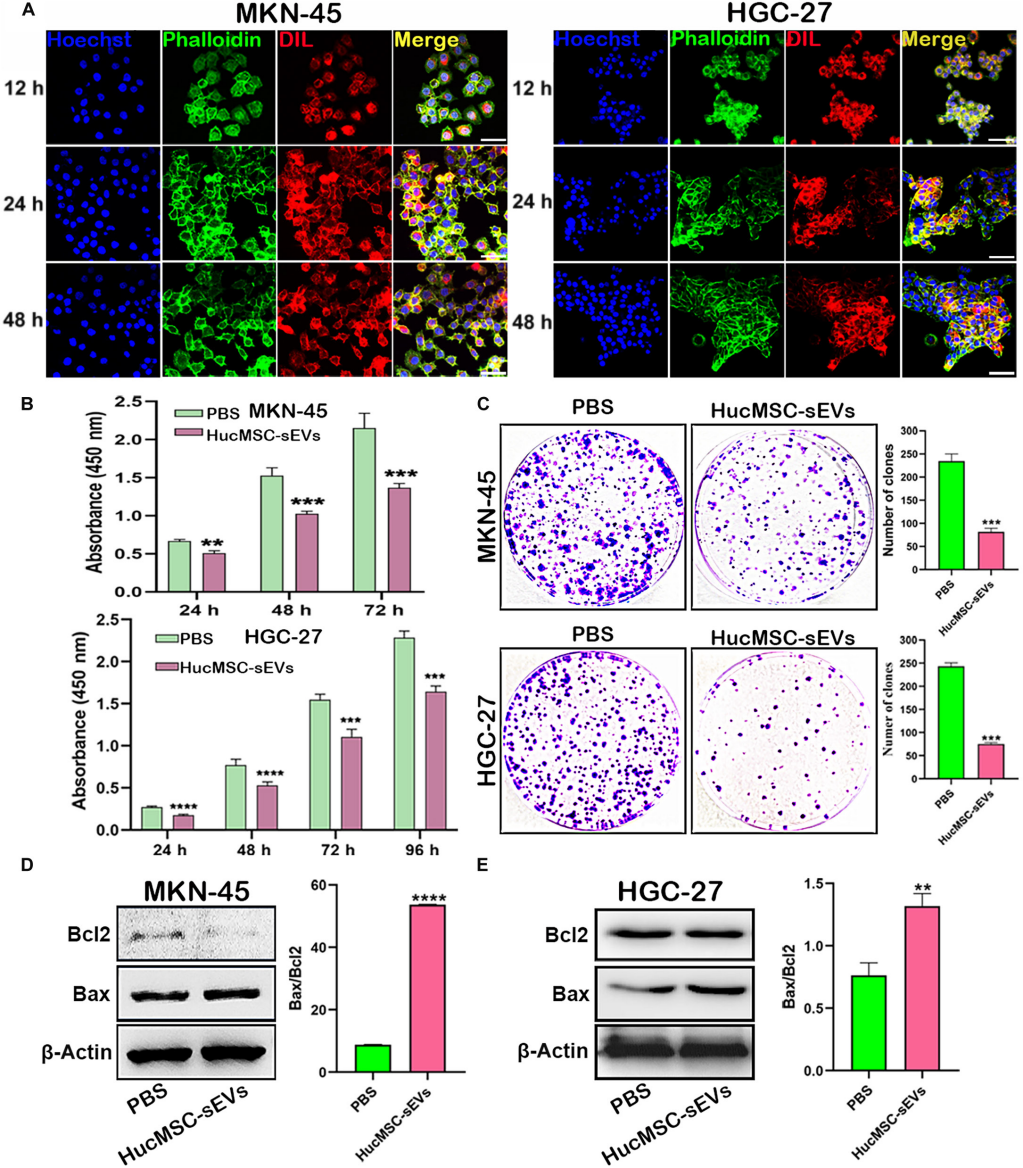
HucMSC-sEVs inhibit GC cell proliferation and promote apoptosis. (A) Confocal microscopy to detect the uptake of DIL-labeled hucMSC-sEVs by the GC cell lines MKN-45 and HGC-27 cells (phalloidin, green fluorescence; DIL, red fluorescence). (B) CCK-8 assay detected the proliferation of GC cell lines MKN-45 and HGC-27 after treatment with hucMSC-sEVs at different time points. (C) The clone formation assay detected cell proliferation of GC cell lines MKN-45 and HGC-27 after treatment with hucMSC-sEVs for 48 h. (D and E) Western blotting to detect apoptosis of GC cell lines MKN-45 and HGC-27 after treatment with hucMSC-sEVs for 48 h.

The P3 generation of hucMSCs underwent identification of the multilineage differentiation potential (Fig. S1A). Adipogenic induction culture of hucMSCs revealed numerous orange-red lipid droplets of varying sizes, round or oval in shape, as visualized by Oil Red O staining (Fig. S1B). Additionally, Alizarin Red S staining demonstrated the presence of characteristic red calcium nodules in cells, indicative of osteogenic differentiation (Fig. S1C). These results confirm the adipogenic and osteogenic differentiation capabilities of hucMSCs. Flow cytometry analysis also demonstrated positive expression of surface markers CD29, CD73, and CD105, and negative expression of markers CD11b, CD14, and CD45 in hucMSCs (Fig. S1D). The sEVs derived from human umbilical cord mesenchymal stem cells (hucMSC-sEVs) were isolated from conditioned medium using a differential ultracentrifugation method established in our laboratory [6]. Nanoparticle tracking analyzer (NTA) revealed that hucMSC-sEVs predominantly ranged in size from 30 to 200 nm, with a peak at 160 nm (Fig. S1E). TEM and AFM images depicted hucMSC-sEVs with a characteristic cup-shaped morphology (Fig. S1F and G). Western blotting analysis confirmed positive expression of the Alix, TSG101, CD63, and CD81 markers, and negative expression of calnexin in hucMSC-sEVs (Fig. S1H). Rich in various proteins and nucleic acids, hucMSC-sEVs can transport bioactive cargoes to tumor cells, effectively inhibiting the malignant progression of various tumors [6,7]. In order to visually trace the entry of hucMSC-sEVs into GC cells, we stained the cytoskeleton with phalloidin and labeled hucMSC-sEVs with DIL. Confocal microscopy examination demonstrated efficient uptake of DIL-labeled hucMSC-sEVs by GC cell lines MKN-45, HGC-27 (Fig. 2A), and SNU-1 (Fig. S2A), with uptake efficiency increasing in a time-dependent manner. Moreover, hucMSC-sEVs are distributed in both cytoplasm and nucleus. Functional assays using CCK-8 and clone formation assays revealed that hucMSC-sEVs inhibited the proliferation of GC cell lines MKN-45, HGC-27 (Fig. 2B and C), and SNU-1 (Fig. S2B and C). Furthermore, Western blotting analysis indicated that hucMSC-sEV treatment effectively promoted GC cell apoptosis MKN-45, HGC-27 (Fig. 2D), and SNU-1 (Fig. S2D). In addition, we found that hucMSC-sEVs at different concentrations had no significant effect on the proliferative activity of GES-1 cells (Fig. S3). Collectively, these findings suggest that hucMSC-sEVs hold promise as natural therapeutic agents for inhibiting the progression of GC.

### HucMSC-sEVs enriched with multiple antitumor molecules

**Fig. 3.**
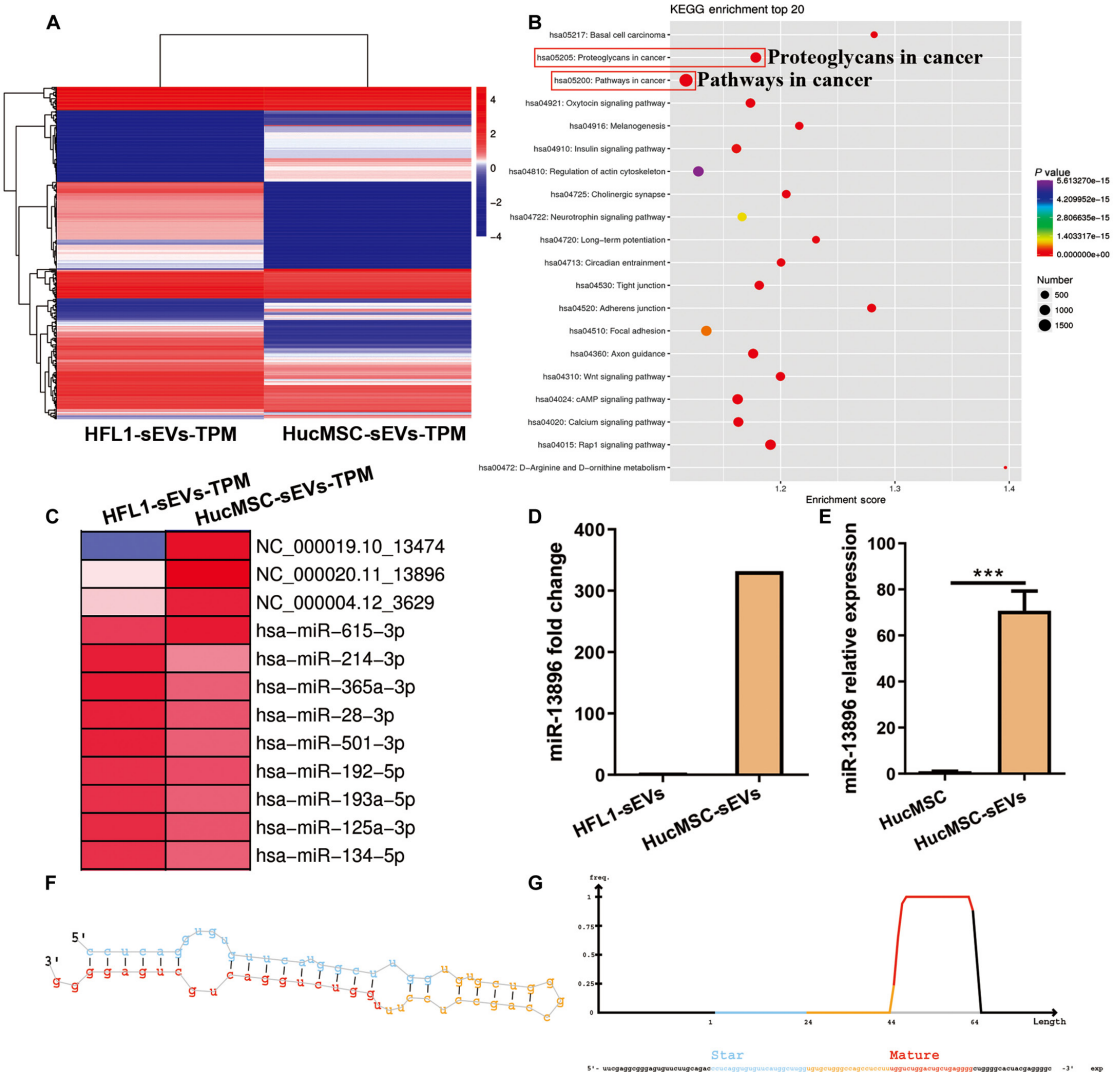
The miRNA microarray revealed enrichment of multiple antitumor molecules in hucMSC-sEVs. (A) MiRNA sequencing screening for differentially expressed miRNAs between HFL1-sEVs and hucMSC-sEVs. (B) KEGG enrichment analysis of the top 20 miRNA-regulated signaling pathways in hucMSC-sEVs. (C) MiRNA sequencing revealed a miR-13896 molecule with significantly high expression in hucMSC-sEVs. (D) Fold change in the miR-13896 expression in sequencing results of HFL1-sEVs and hucMSC-sEVs. (E) qRT-PCR detection of miR-13896 expression in hucMSC-sEVs and hucMSCs. (F and G) Molecular precursor and mature structure of miR-13896.

To elucidate the mechanism by which sEVs derived from hucMSC-sEVs (hucMSC-sEV) inhibit malignant progression of GC, we performed miRNA sequencing in 3 different batches of HFL1-sEVs and hucMSC-sEVs. Comparative analysis revealed that hucMSC-sEVs exhibited differential expression of miRNAs compared to HFL1-sEVs, with 102 miRNAs up-regulated and 220 miRNAs down-regulated in hucMSC-sEVs. In particular, hucMSC-sEVs contained 14 known anticancer miRNAs, including miR-122, miR-124, miR-143, miR-145, and miR-375 (Fig. 3A). Furthermore, the Kyoto Encyclopedia of Genes and Genomes (KEGG) enrichment analysis of the top 20 signaling pathways indicated that the miRNAs enriched in hucMSC-sEVs are closely associated with tumor regulation (Fig. 3B). In addition, novel miRNAs were identified through sequencing. Of these, 71 miRNAs were detected in HFL1-sEVs, whereas hucMSC-sEVs harbored 84 miRNAs. Particularly noteworthy were the significant up-regulation of miRNAs in hucMSC-sEVs, such as NC_000011.10_8535, NC_000022.11_14216, NC_000019.10_13474, NC_000020.11_13896 (miR-13896), and NC_000014.9_10228, with fold changes of 3,470, 3,095, 1,740, 332, and 6, respectively (Fig. 3C). Subsequent qRT-PCR and functional experiments preliminarily validated miR-13896 as a potential tumor suppressor molecule. Detailed analyses of precursor and mature forms of miR-13896, along with its expression levels in sEVs and hucMSCs, demonstrated substantial enrichment in hucMSC-sEVs compared to HFL1-sEVs and hucMSCs (Fig. 3D to G). These findings underscore the crucial role of miRNA cargo in hucMSC-sEVs that mediate their inhibitory effects on GC progression.

### miR-13896 inhibited the proliferation and metastasis of GC cells and promoted their apoptosis

**Fig. 4.**
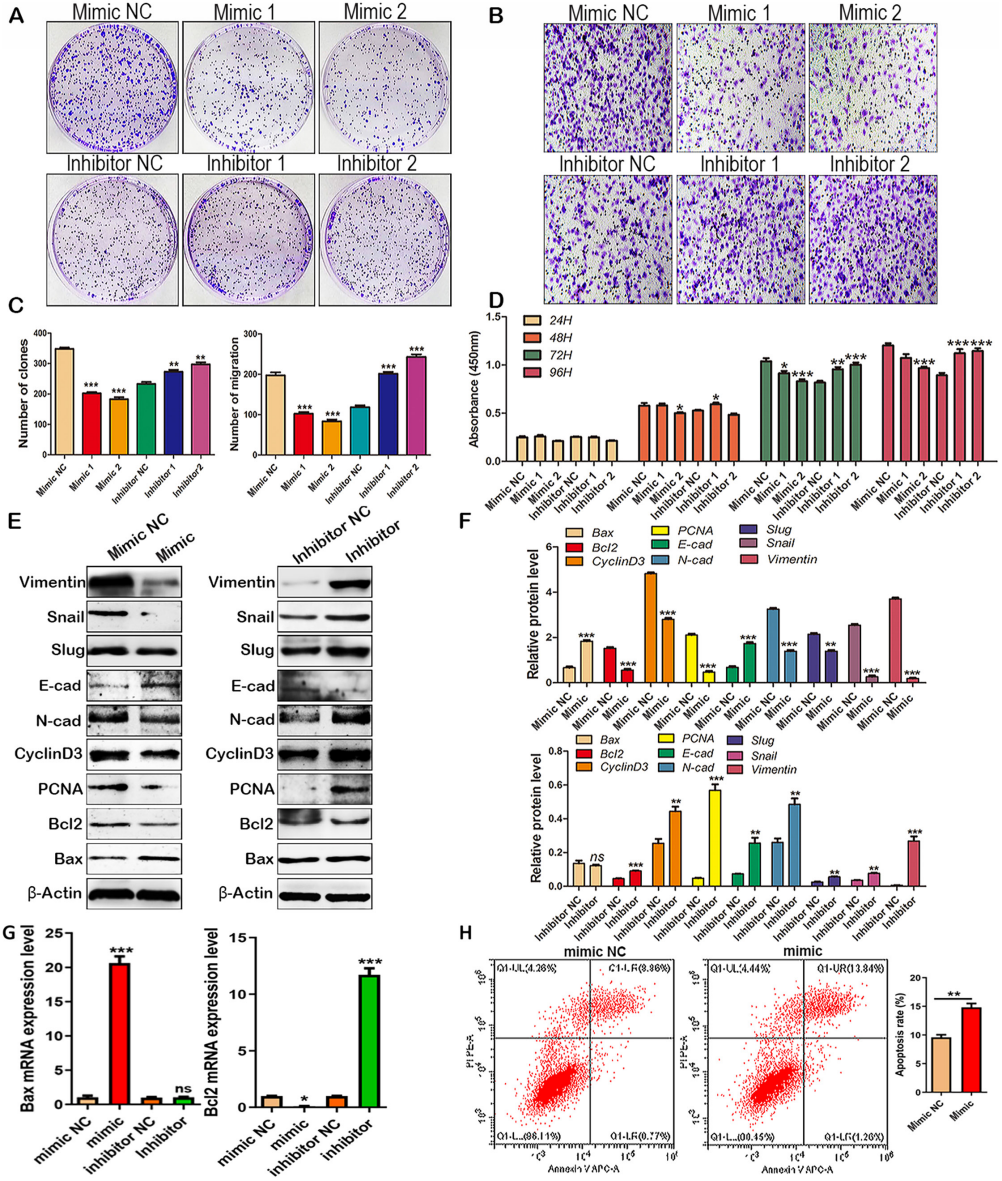
miR-13896 inhibits the proliferation and metastasis of MKN-45 cells. (A) A colony formation assay was performed to detect colony formation of MKN-45 cells transfected with different concentrations of miR-13896 mimic and inhibitor (2.5 and 5 nmol) for 48 h. (B) The Transwell assay was performed to detect the migration of MKN-45 cells transfected with different concentrations of miR-13896 and inhibitor for 48 h. (C) Cell counts in (A) and (B). (D) The CCK-8 assay was performed to detect proliferation of MKN-45 cells transfected with different concentrations of miR-13896 mimic and inhibitor for 48 h. (E) Western blotting was performed to detect the expression of proteins related to proliferation, migration, and apoptosis in MKN-45 cells transfected with 5 nmol concentrations of miR-13896 mimic and inhibitor for 48 h. (F) Densitometric analysis of protein bands in Fig. 3E. (G) qRT-PCR was performed to detect the expression of genes in MKN-45 cells transfected with 5 nmol concentrations of miR-13896 mimic and inhibitor for 48 h. (H) Flow cytometry was used to detect the apoptosis of MKN-45 cells transfected with 5 nmol concentration of miR-13896 mimic for 48 h.

MiRNA mimics are chemically synthesized mature miRNA mimics that mimic endogenous miRNAs and specifically enhance the function of endogenous miRNA. miRNA inhibitor is a chemically modified miRNA inhibitor, which is specialized for specific target miRNA inhibitors in cells, and can specifically and efficiently inhibit the activity of endogenous miRNA in organisms. To verify the function of miR-13896 in GC inhibition, we commissioned Gemma gene to design and synthesize mimics and inhibitors of miR-13896. The colony formation assay demonstrated that overexpression of miR-13896 in GC cells significantly suppressed the colony formation capacity of MKN-45 cells, whereas inhibition of miR-13896 expression promoted colony formation (Fig. 4A). Similarly, the Transwell migration assay showed that miR-13896 overexpression inhibited the migration ability of MKN-45 cells, whereas inhibition of miR-13896 enhanced migration (Fig. 4B). Consistent with these findings, the CCK-8 proliferation assay confirmed that miR-13896 overexpression attenuated MKN-45 cell proliferation (Fig. 4D). Western blotting analysis further elucidated the molecular mechanisms underlying miR-13896 activity. Overexpression of miR-13896 in GC cells decreased key proliferation-related genes, including PCNA and Cyclin D3, and the antiapoptotic gene Bcl2, while up-regulating that of the apoptosis-promoting gene Bax. Furthermore, overexpression of miR-13896 inhibited the expression of migration-related genes such as vimentin, Snail, Slug, and N-cadherin, and increased the expression of the migration-suppressing gene E-cadherin in MKN-45 cells (Fig. 4E). Conversely, inhibition of miR-13896 expression exerted opposite effects on these genes in MKN-45 cells (Fig. 4E). qRT-PCR analysis validated these findings, showing that miR-13896 overexpression promoted Bax mRNA expression and suppressed Bcl2 mRNA expression in GC cells, whereas miR-13896 had the opposite effect (Fig. 4G). Flow cytometry analysis further confirmed that miR-13896 overexpression induced apoptosis in MKN-45 cells (Fig. 4H). Furthermore, miR-13896 also inhibited self-renewal capacity, as evidenced by the reduced expression of stem-associated transcription factors Oct4 and Sox2 in MKN-45 cells (Fig. S4A and B). In addition, we also found that miR-13896-engineered hucMSC-sEVs also significantly inhibited the proliferation of HGC-27 and SNU-1 cells and promoted their apoptosis (Fig. S5). In addition, we examined the effects of hucMSC-sEVs engineered by miR-13896 on normal gastric mucosal epithelial cells after GES-1 intervention. The results of cell viability test and colony formation assay showed that miR-13896 mimic-engineered hucMSC-sEVs had no significant effect on GES-1 cell proliferation (Fig. S6). However, miR-13896 inhibitor overexpression of hucMSC-sEVs significantly inhibited GES-1 cell proliferation (Fig. S6). This suggests that the expression of miR-13896 by GES-1 cells is necessary for their own survival. These comprehensive findings collectively highlight miR-13896, delivered by hucMSC-sEVs, as a novel anticancer molecule capable of suppressing GC progression.

### miR-13896 inhibited the autophagy signaling pathway in GC cells

**Fig. 5.**
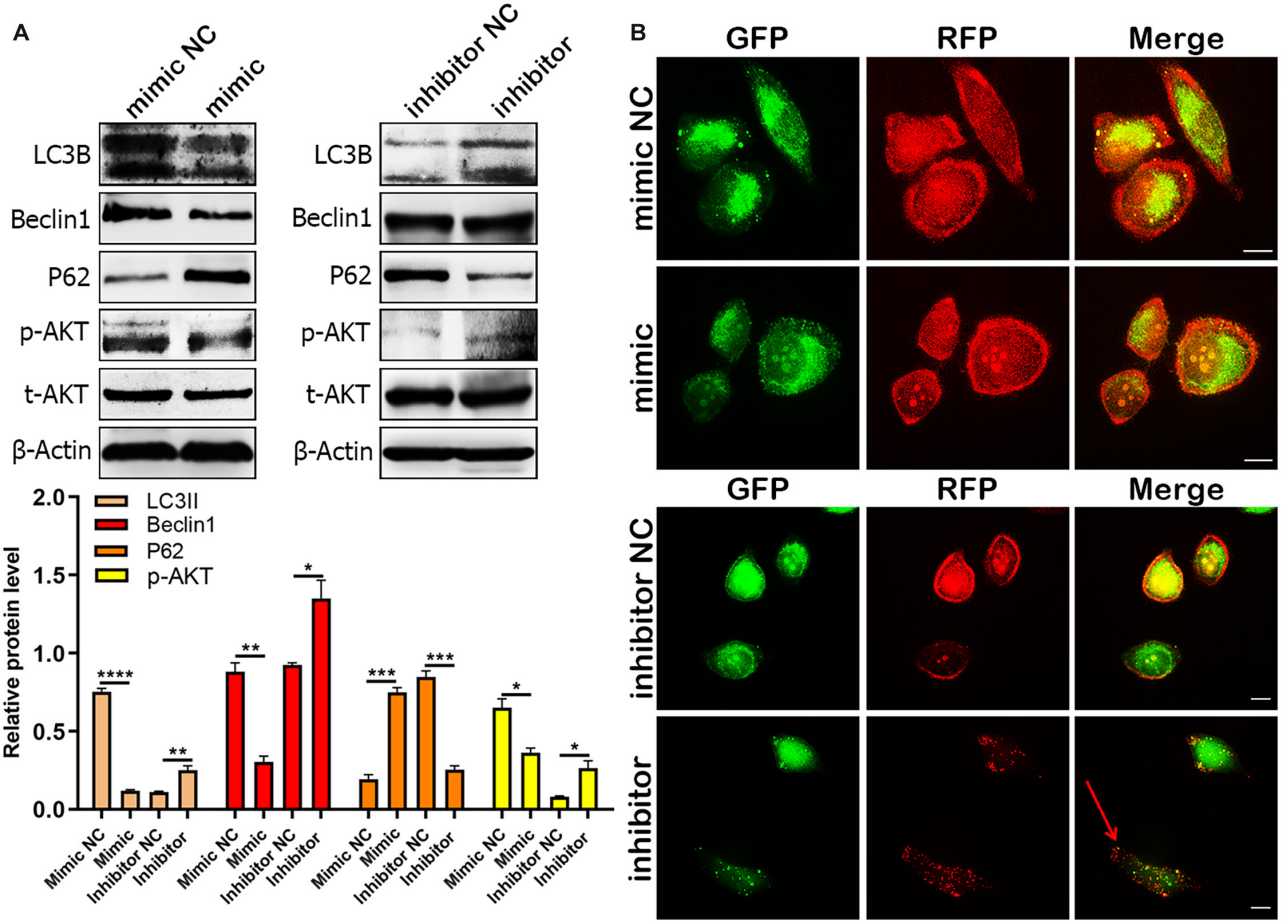
miR-13896 inhibits autophagy activation in GC cells. (A) Western blotting was used to detect the expression of autophagy-related proteins in MKN-45 cells transfected with 5 nM miR-13896 mimic and inhibitor for 48 h. (B) Confocal microscopy was used to detect autophagy flow in MKN-45 cells transfected with 5 nM miR-13896 mimic and inhibitor for 48 h.

Autophagy has been implicated in the pathogenesis of GC, which led to our investigation into the mechanistic role of miR-13896 in tumor suppression through its influence on cell autophagy. Using miRNA sequencing and the prediction of the target gene of hucMSC-sEVs, we identified multiple target proteins for miR-13896, including the autophagy-related protein ATG2A. This led us to hypothesize that miR-13896 exerts its tumor-suppressing effects by modulating the autophagy signaling pathway. Western blotting analysis revealed that miR-13896 overexpression negatively regulated the expression of phosphorylated AKT (p-AKT) in GC cells, simultaneously suppressing the levels of autophagy-associated proteins LC3B and Beclin1 while improving the expression of the autophagy inhibitor P62 (Fig. 5A). To further explore this, autophagy double-labeling adenovirus assays demonstrated that miR-13896 overexpression inhibited autophagosome formation, whereas suppression of miR-13896 expression promoted autophagosome formation (Fig. 5B and Fig. S7).

### miR-13896 binds and inhibits the expression of the autophagy-related protein ATG2A in GC cells

**Fig. 6.**
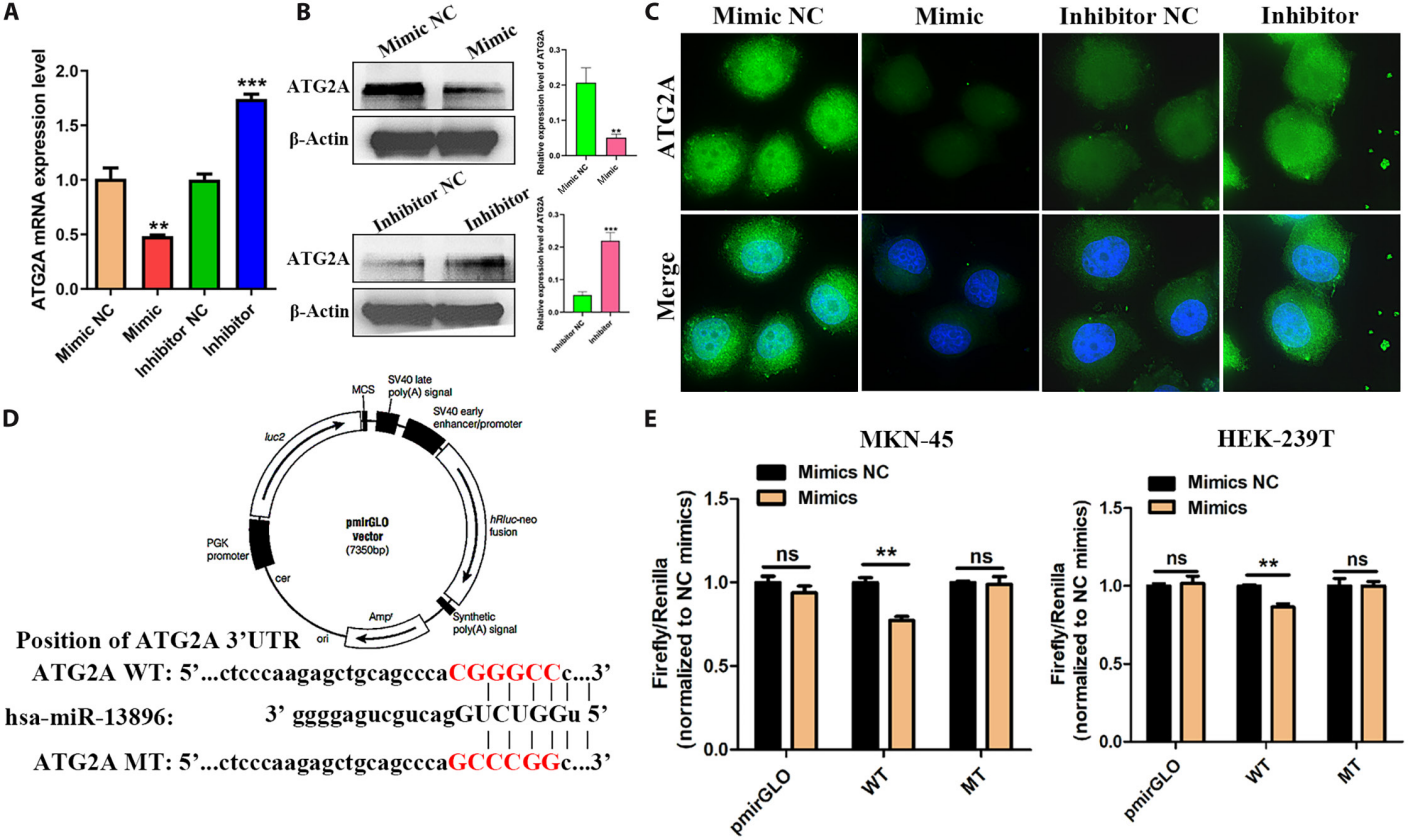
miR-13896 targets and inhibits the expression level of ATG2A. (A) qRT-PCR was used to detect changes in ATG2A mRNA after 48 h of transfection of 5 nmol concentration of miR-13896 mimic and inhibitor into MKN-45 cells. (B and C) Western blotting and IF were used to detect changes in the expression of ATG2A protein after 48 h of transfection of 5 nmol concentration of miR-13896 mimic and inhibitor into MKN-45 cells. (D) Bioinformatics software was used to predict the binding sites of miR-13896 and ATG2A. (E) A dual-luciferase reporter gene assay was used to detect luciferase expression after cotransfection of miR-13896 and ATG2A plasmids into MKN-45 and HEK-293T cells.

In order to explore the molecular mechanism of miR-13896 inhibiting autophagy, we examined its effect on ATG2A protein in GC cells. Subsequently, we investigated whether miR-13896 regulates ATG2A to modulate autophagy levels in GC cells. qRT-PCR, Western blotting, and IF assays collectively demonstrated that overexpression of miR-13896 suppressed both mRNA and protein levels of ATG2A in MKN-45 cells (Fig. 6A to C). To validate the interaction between miR-13896 and ATG2A, we used bioinformatics tools to predict potential binding sites and constructed a dual-luciferase reporter plasmid (Fig. 6D). Dual-luciferase reporter assays confirmed that miR-13896 bound directly to ATG2A and reduced its expression in MKN-45 and HEK-293T cells (Fig. 6E).

### The ATG2A was overexpressed in GC tissues and cells and was positively correlated with the level of autophagy

**Fig. 7.**
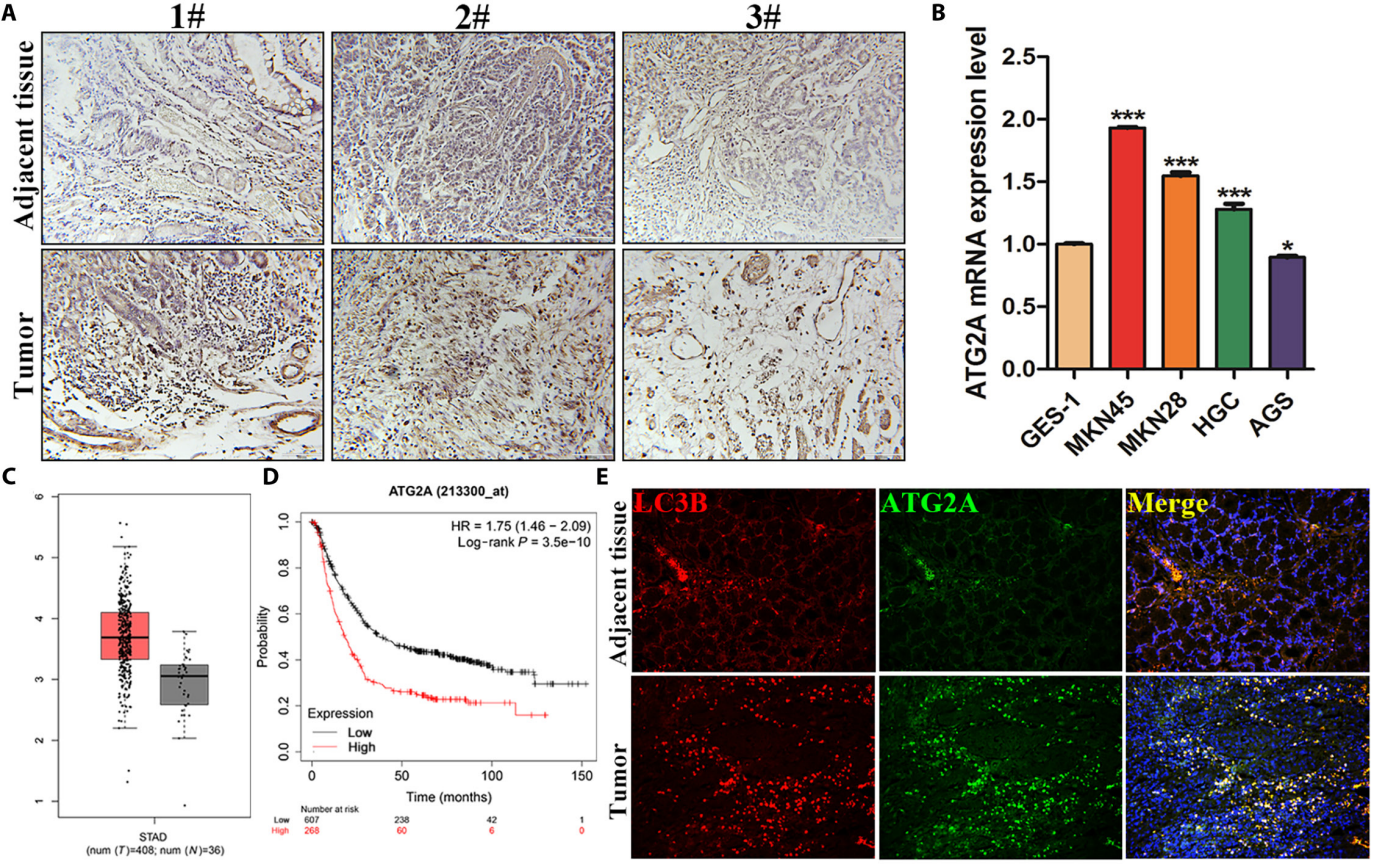
ATG2A expression is elevated in GC tissues and cells and is positively correlated with autophagy. (A) Immunohistochemical staining showing the expression of ATG2A in tumor and adjacent tissues (200×). (B) qRT-PCR was used to detect the expression of ATG2A in normal gastric epithelial cells GES-1 and GC cell lines. (C and D) Bioinformatics analysis of ATG2A expression and survival curve in normal and tumor patients. (E) Colocalization of ATG2A and LC3B in tumor and adjacent tissues detected by IF (200×).

To delineate the role of ATG2A in GC pathogenesis, we evaluated its expression in GC tissues and cell lines. IHC analysis revealed markedly increased ATG2A expression in tumor tissues compared to adjacent nontumor tissues from patients with GC (Fig. 7A). qRT-PCR also confirmed a markedly higher expression of ATG2A in GC cell lines relative to normal gastric mucosal epithelial cells (GES-1) (Fig. 7B). Analysis using the GEPIA2 database corroborated these findings, demonstrating higher expression of ATG2A in tumor samples compared to healthy controls (Fig. 7C). Kaplan–Meier survival curve analysis indicated that patients with GC with elevated ATG2A expression exhibited a poorer overall prognosis compared to those with lower expression (Fig. 7D). IF staining illustrated robust coexpression and colocalization of ATG2A with the autophagy marker LC3B in tumor tissues of the GC (Fig. 7E).

### Silencing ATG2A expression in GC cells inhibited the proliferation of GC cells and promoted their apoptosis by inhibiting autophagy

**Fig. 8.**
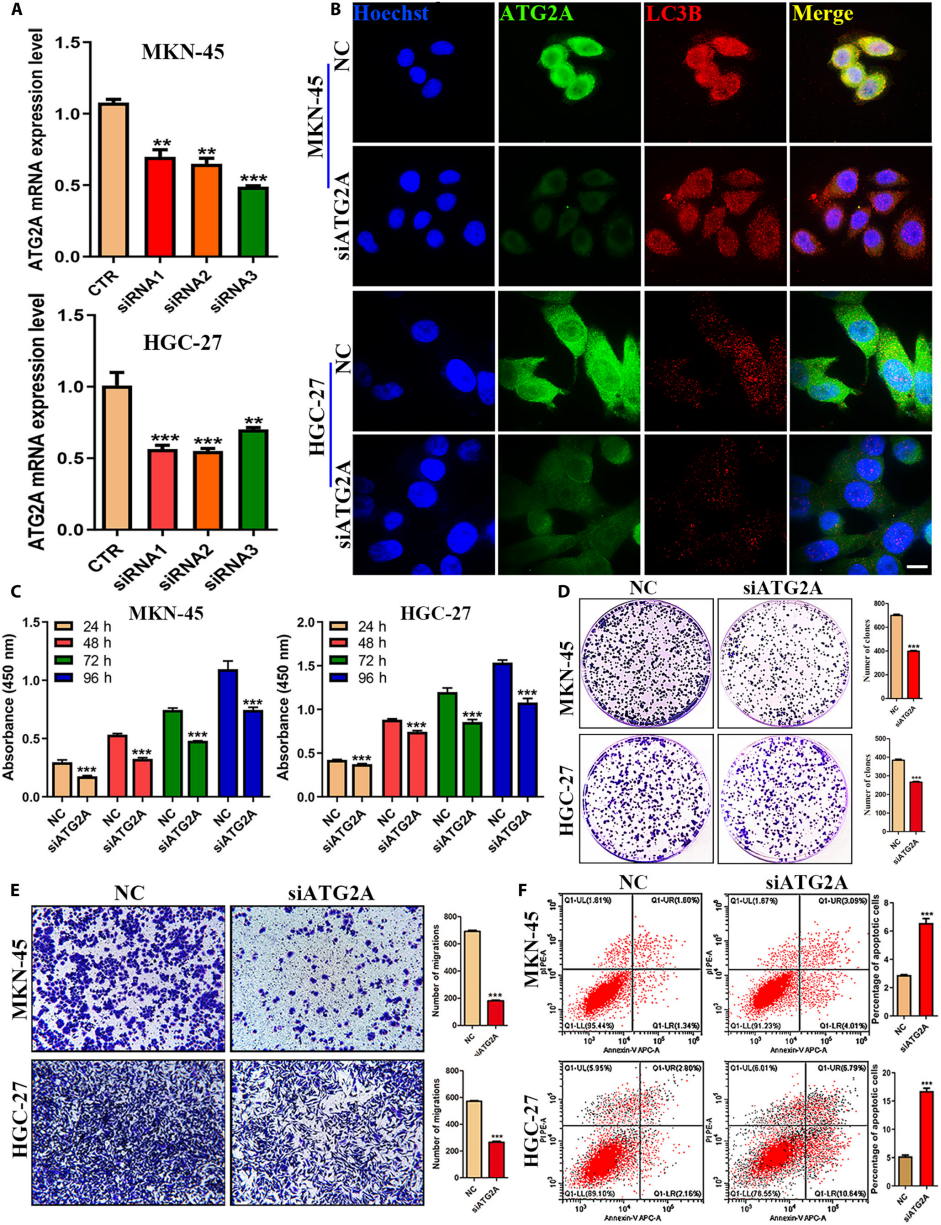
ATG2A knockdown significantly inhibits proliferation and promotes apoptosis of GC cells. (A) qRT-PCR was used to detect the level of ATG2A in MKN-45 and HGC-27 cells after transfection of 3 ATG2A-siRNA fragments for 48 h. (B) Protein expression of ATG2A in MKN-45 and HGC-27 cells detected 48 h after transfection with siRNA3 and siRNA2 fragments into MKN-45 and HGC-27 cells, respectively (600×). (C) Findings of the CCK-8 assay showing the proliferation activity of MKN-45 and HGC-27 cells after transfection of ATG2A-siRNA3 and siRNA2 fragments for 48 h. (D) Colony formation of MKN-45 and HGC-27 cells after transfection of ATG2A-siRNA3 and siRNA2 fragments for 48 h was detected by clone formation assay. (E) Transwell assay to determine cell migration of ATG2A-siRNA3 and siRNA2 fragments after transfection into MKN-45 and HGC-27 cells for 48 h (100×). (F) Apoptosis of MKN-45 and HGC-27 cells 48 h after transfection of fragments of ATG2A-siRNA3 and siRNA2 into MKN-45 and HGC-27 cells detected by flow cytometry.

To elucidate the role of ATG2A in GC cells, we used siRNA-mediated silencing of ATG2A in MKN-45 and HGC-27 cell lines. qRT-PCR analysis confirmed effective silencing of ATG2A by the 3 siRNA fragments tested. Specifically, siRNA3 demonstrated superior knockdown efficiency in MKN-45 cells, while siRNA2 exhibited better efficacy in HGC-27 cells (Fig. 8A). IF staining further substantiated these findings, revealing markedly reduced expression of ATG2A tagged with green fluorescent protein in MKN-45 and HGC-27 cells transfected with siRNA fragments after 48 h. This reduction was correlated with the down-regulation of the autophagy marker LC3B (Fig. 8B). Subsequent to elucidating the role of ATG2A in the regulation of autophagy in GC, we investigated its functional impact on GC cells. The results of CCK-8 assays, colony formation assays, Transwell migration assays, and apoptosis staining experiments collectively demonstrated that silencing the ATG2A protein in GC cells substantially attenuated its proliferation, colony formation capacity, and migratory potential while concomitantly improving apoptotic rates (Fig. 8C to F).

### Preparation and characterization of miR-13896-engineered hucMSC-sEVs

**Fig. 9.**
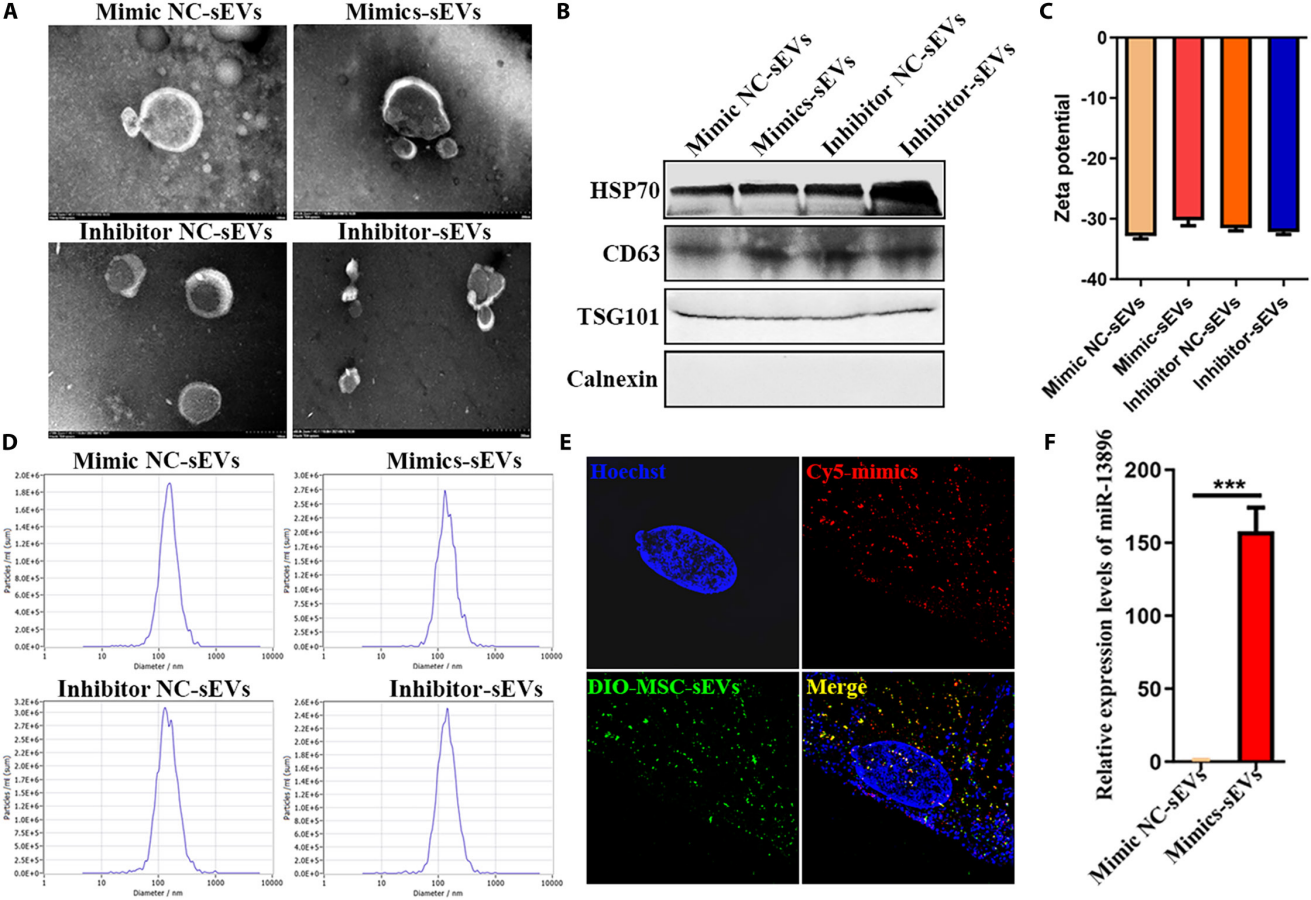
Preparation and characterization of miR-13896-engineered hucMSC-sEVs. (A) TEM detection of the size and morphology of miR-13896-engineered hucMSC-sEVs. (B) Western blotting detection of surface markers of miR-13896-engineered hucMSC-sEVs. (C and D) NTA detection of the particle size, concentration, and membrane potential of miR-13896-engineered hucMSC-sEVs. (E) Super-resolution microscopy detection of the uptake of miR-13896-engineered hucMSC-sEVs by the GC cell line MKN-45. (F) qRT-PCR detection of the loading efficiency of the electroporation-loading exogenous miR-13896 mimic in hucMSC-sEVs.

To augment the inhibitory efficacy of hucMSC-sEVs on GC cells, we employed electroporation technology to incorporate exogenous miR-13896 mimic and inhibitor into hucMSC-sEVs. TEM results indicated that electroporation did not alter the morphology of hucMSC-sEVs, as all 4 groups of hucMSC-sEVs maintained their characteristic “cup-shaped” structure (Fig. 9A). Western blotting analysis confirmed that after miR-13896 loading, hucMSC-sEVs retained characteristic sEV-positive surface markers, such as CD63, HSP70, and TSG101, while lacking expression of the negative surface marker calnexin (Fig. 9B). NTA demonstrated that electroporation had a minimal impact on membrane potential and particle size distribution of hucMSC-sEVs (Fig. 9C and D). To assess loading efficiency, the Cy5-labeled miR-13896 mimic was electroporated into DIO-labeled hucMSC-sEVs. Ultra-high-resolution microscopy confirmed that miR-13896-engineered hucMSC-sEVs efficiently internalized into MKN-45 cells, with Cy5-labeled miR-13896 mimic and DIO-labeled hucMSC-sEVs colocalizing in the nucleus and cytoplasm (Fig. 9E), underscoring the effective loading of miRNA without compromising the integrity of the sEVs. Furthermore, qRT-PCR analysis revealed that the expression of miR-13896 in electroporated hucMSC sEVs was nearly 200-fold higher than that of natural hucMSC- sEVs (Fig. 9F).

### miR-13896-engineered hucMSC-sEVs inhibited GC growth in vitro and in vivo

**Fig. 10.**
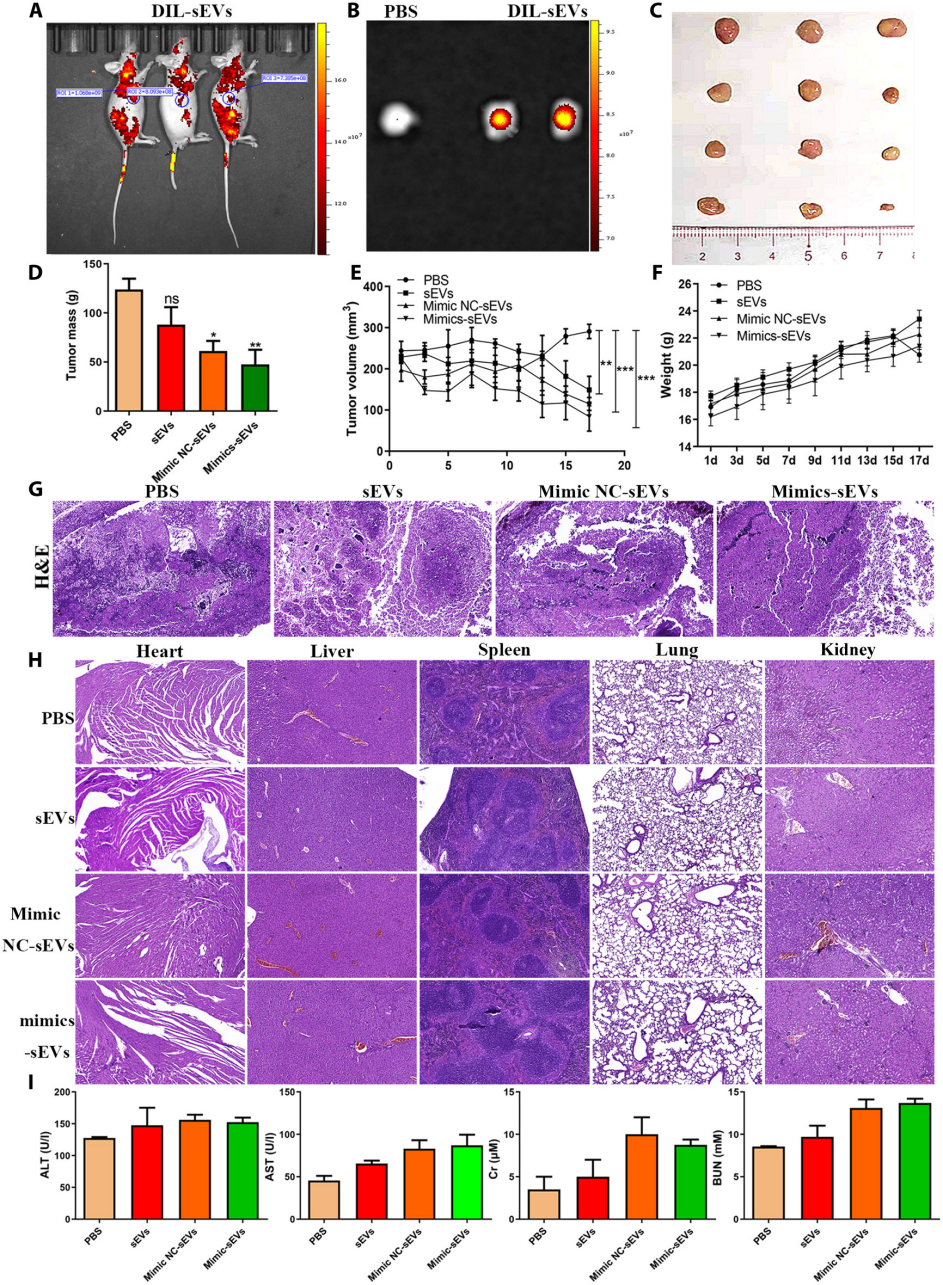
Inhibition of GC growth by miR-13896-engineered hucMSC-sEVs in vivo. (A) Small-animal in vivo imaging detection of tumor site imaging after tail vein injection of DIL-labeled hucMSC-sEVs to subcutaneous tumor-bearing mice for 24 h. (B) Small-animal in vivo imaging detection of tumor in vitro tissue imaging after tail vein injection of DIL-labeled hucMSC-sEVs to subcutaneous tumor-bearing mice for 24 h. (C) Gross view of tumor after miR-13896-engineered hucMSC-sEV treatment in tumor-bearing mice. (D) Statistical results of tumor mass in each group of mice. (E) Continuous monitoring of tumor volume growth in miR-13896-engineered hucMSC-sEV treatment in tumor-bearing mice for 20 d. (F) Continuous monitoring of body weight after miR-13896-engineered hucMSC-sEV treatment in tumor-bearing mice for 20 d. (G) H&E staining detection of histological changes in GC after the end of miR-13896-engineered hucMSC-sEV treatment (40×). (H) H&E staining to detect structural changes in main organs in tumor-bearing mice treated with miR-13896-engineered hucMSC-sEVs. (I) Biochemical experiments were performed to detect changes in liver and kidney function in tumor-bearing mice treated with miR-13896-engineered hucMSC-sEVs.

Following the successful construction of miR-13896-engineered hucMSC-sEVs, their tumor-suppressing effects were evaluated in vitro. Colony formation and CCK-8 assays revealed that the group treated with miR-13896 mimic-overexpressing hucMSC-sEVs significantly suppressed GC cell proliferation compared to the mimic NC group, whereas the group treated with inhibitor-loaded hucMSC-sEVs promoted GC cell proliferation (Fig. S8A to C). Moreover, Western blotting analysis demonstrated that miR-13896 mimic-overexpressing hucMSC-sEVs markedly down-regulated the autophagy markers LC3B and P62 in GC cells, whereas inhibitor-loaded hucMSC-sEVs enhanced autophagy levels (Fig. S8D). In vivo imaging of small animals showed that DIL-labeled miR-13896-engineered hucMSC-sEVs actively targeted and accumulated at tumor sites (Fig. 10A and B). Following a 20-day treatment regimen, the miR-13896-engineered hucMSC-sEV-treated group exhibited substantially reduced tumor weight and volume (Fig. 10C to E), without any observable effect on body weight (Fig. 10F). Histopathological examination via H&E staining revealed severe necrosis, changes in cell vacuolation, and inflammatory cell infiltration in the miR-13896-engineered hucMSC-sEV-treated group compared to the PBS-treated group (Fig. 10G). Given that hucMSC-sEVs have been reported to be safe and effective therapeutic agents for tissue regeneration and tumor suppression in vitro and in vivo, we further evaluated the safety of miR-13896-engineered hucMSC-sEVs prepared using electroporation-loading technology for GC treatment in vivo. After treatment, the main organs (heart, liver, spleen, lung, and kidney) were collected for histopathological evaluation. H&E staining did not show significant alterations in tissue structure in the PBS, hucMSC-sEVs, miR-13896 NC-sEVs, or miR-13896 mimic-sEVs groups (Fig. 10H). Furthermore, serological analysis indicated no significant changes in liver or kidney function indicators (aspartate aminotransferase, alanine aminotransferase, creatinine, or blood urea nitrogen) between the intervention groups (Fig. 10I).

GC remains a significant global health challenge due to its high mortality rate and substantial economic burden on individuals, families, and society. Effective strategies for early diagnosis, prevention, and treatment are urgently needed to mitigate these impacts. MSCs have emerged as promising candidates for cancer therapy, leveraging their inherent tumor-homing capabilities and multifaceted antitumor mechanisms. These mechanisms include inhibition of angiogenesis, modulation of inflammatory cell infiltration, promotion of tumor cell apoptosis, cell cycle arrest, and suppression of pro-proliferative signaling pathways such as AKT and WNT activation [16]. HucMSCs have demonstrated superior safety and efficacy in tumor suppression compared to MSCs derived from bone marrow or adipose tissue. A significant body of evidence supports their ability to inhibit the progression of various cancers through direct cell contact or paracrine actions [17–21]. Despite these findings, the precise molecular mechanisms underlying their antitumor effects remain incompletely understood.

EVs, specifically those derived from MSCs, have garnered attention as potent nanocarriers for therapeutic cargo. These bilayer membranous vesicles, ranging from 30 to 200 nm in diameter, play a pivotal role in mediating the biological functions of MSCs in tumor suppression. EVs can transfer genetic information or proteins and metabolic components of the mother cell to the target cell through various ways such as membrane fusion, ligand-mediated, and endocytosis and pinocytosis, thus playing an important role [7]. MSC-sEVs of various tissue origins have been shown to inhibit tumor progression by delivering tumor suppressor miRNAs to cancer cells. For example, bone marrow-derived MSC-sEVs (BMMSC-sEVs) have been implicated in various antitumor mechanisms. Zhang et al. [22] demonstrated that BMMSC-sEVs delivered miR-206 to target TRA2B (transformer 2β), inhibiting osteosarcoma progression. Similarly, Cai et al. [23] found that BMMSC-sEVs containing miR-9-3p suppressed bladder cancer growth and metastasis by targeting ESM1 (endothelial cell specific molecule 1). Moreover, studies by Pakravan et al*.* [24] and Lee et al. [25] also proved that BMMSC-sEVs carrying miR-100 could regulate the mammalian target of rapamycin (mTOR)/hypoxia-inducible factor-1α (HIF-1α)/vascular endothelial growth factor (VEGF) signaling axis and inhibit angiogenesis of breast cancer cells in vitro, while sEVs carrying miR-16 could inhibit angiogenesis by down-regulating VEGF expression in breast cancer cells. Similarly, BMMSC-sEVs containing miR-23b can also promote the dormancy of metastatic breast cancer cells by targeting MARCKS (myristoylated alanine-rich C kinase substrate) protein expression [26]. Beyond BMMSCs, sEVs derived from adipose tissue, umbilical cord, uterine blood, and embryonic stem cells have also shown anticancer effects through various mechanisms. Takahara et al. [27] found that adipose MSC-sEVs containing miR-145 inhibited prostate cancer cell growth by increasing the expression of caspase 3/7 expression and reducing the expression of the anti-apoptotic protein Bcl-xL. Adipose MSC-sEVs delivered miR-122 to regulate CCNG1, IGF1R, and ADAM10 molecules to increase sensitivity to chemotherapy of hepatocellular carcinoma [28]. HucMSC-EVs mediated the increased sensitivity of human leukemia cells K562 to imatinib by activating the caspase signaling pathway, and played a significant anti-leukemia effect [29]. In addition, HucMSC-EVs promoted imatinib-induced apoptosis in K562-R cells via a miR-145a-5p/USP6/GLS1 axis [30]. He et al. [31] reported that hucMSC-sEVs delivered miR-375 targeting ENAH to block the progression of esophageal squamous cell carcinoma. Rosenberger et al. [32] demonstrated that human uterine blood MSC-sEVs inhibited angiogenesis and tumor growth in oral squamous cell carcinoma, while Zhu et al. [33] found that embryonic stem cell-derived sEVs could be used for the treatment of glioblastoma. However, the specific molecular pathways governing these anticancer effects require further elucidation. In conclusion, MSC-derived sEVs represent a promising avenue for developing novel therapeutic strategies against GC and other cancers. Their ability to deliver bioactive molecules, including miRNAs, to tumor cells underscores their potential in precision medicine. Future research efforts should focus on unraveling the intricate molecular mechanisms of antitumor activities of MSC-sEVs to optimize their clinical application and advance cancer treatment paradigms.

Our study demonstrates that hucMSC-sEVs efficiently enter GC cells in a time-dependent manner, effectively inhibiting cell proliferation and metastasis and promoting apoptosis. sEVs, particularly enriched with miRNAs, have emerged as critical mediators of these antitumor effects, surpassing their parent cells in miRNA composition and therapeutic potential [34]. Enriched miRNAs in MSC-sEVs play an important role in mediating their antitumor effects. To explore the potential mechanism of hucMSC-sEVs in inhibiting GC, we performed miRNA sequencing on 3 batches of HFL1-sEVs and hucMSC-sEVs to detect differentially expressed miRNA molecules involved in inhibition of GC. Compared to HFL1-sEVs, 102 miRNAs were up-regulated and 220 miRNAs were down-regulated in hucMSC-sEVs, including 14 miRNA molecules confirmed to have anticancer activity, such as miR-122, miR-124, miR-143, miR-145, and miR-375. KEGG enrichment analysis showed that the enriched miRNAs in hucMSC-sEVs were closely related to tumor regulation. Furthermore, 84 previously undescribed miRNAs were also found in hucMSC-sEVs. These molecules with significant up-regulation included NC_000011.10_8535, NC_000022.11_14216, NC_000019.10_13474, and NC_000020.11_13896 (miR-13896). Subsequently, qRT-PCR experiments also confirmed that these molecules were up-regulated. These results suggest that the GC inhibition effect by hucMSC-sEVs may be closely related to the miRNA molecules carried by them. Through the selection and validation of novel miRNAs up-regulated up in hucMSC-sEVs, miR-13896 was found to be a potential tumor suppressor molecule. Subsequently, the precursor and mature structure of miR-13896 and the molecular expression in sEV and hucMSC cells were detected. Compared with HFL1-sEVs and hucMSCs, hucMSC-sEVs showed significant enrichment. Further functional verification found that miR-13896 is a potential GC inhibitory molecule. In vitro cell experiments showed that overexpression of miR-13896 in GC cells could inhibit the proliferation and migration of GC cells and promote the apoptosis of GC cells.

Autophagy plays an important role in the occurrence, development, metastasis, drug resistance, and immune escape of GC. The autophagy activity of most tumors is altered, and tumor cells use autophagy to promote their survival under nutritional deficiency and hypoxia. Therefore, targeted regulation of autophagy is an important means of tumor therapy. Tang et al*.* [10] also showed that loss of ATG2A/B would transform cell-protective autophagy into activation of atypical caspase-8 and apoptosis. Furthermore, ATG2A interacts with a variety of proteins and mediates changes in autophagy activity, which contribute to the occurrence and development of a variety of tumors [11–13]. Our results suggest that overexpression of miR-13896 in GC cells can reduce the expression level of ATG2A and thus inhibit the autophagy activity of GC cells. The results of the dual-luciferase reporter gene showed that the miR-13896 and ATG2A protein could bind and reduce the expression of ATG2A. Additionally, we also found that ATG2A was significantly overexpressed in the cancer tissues of GC patients, and the prognosis of GC patients with high expression of ATG2A was poor. Silencing of ATG2A significantly inhibited proliferation and promoted apoptosis. These findings further suggest that ATG2A is expected to be an important target for the precision treatment of GC.

MSCs have active homing and chemotaxis characteristics, and sEVs, as the main component of paracrine, have similar characteristics. Therefore, enriching the effective components of antitumor therapy into hucMSC-sEVs is the main research direction for current engineering approaches. The lipid bilayer structure of sEVs provides excellent stability and biocompatibility, which can protect their internal bioactive molecules from degradation. The nanosized membrane structure of sEVs allows them to freely pass through physiological barriers such as the blood–brain barrier to reach the target site. Therefore, hucMSC-sEVs are expected to become an important natural reagent and superior delivery carrier for drug delivery and bioactive molecules, thus inhibiting the malignant progression of GC [35]. In recent years, researchers have found that sEVs engineered and modified by physical, chemical, biological, and other means have significant effects in targeted tumors and drug delivery, and engineered sEVs have become a new means to improve the efficacy. These characteristics also make it an ideal nanodrug delivery carrier [36]. Compared with incubation, ultrasound, extrusion, and other technologies, electroporation technology has been proven to be the most efficient means of engineering exogenous small nucleotides into sEVs. Given their natural biocompatibility and stability conferred by lipid bilayer membranes, hucMSC-sEVs present an ideal platform for delivering bioactive molecules to GC and other tumor sites. Our findings support the potential of engineered hucMSC-sEVs as effective delivery vehicles for enhancing the therapeutic efficacy of miR-13896 in the treatment of patients with GC. Notably, electroporation technology enabled efficient loading of exogenous miR-13896 mimics into hucMSC-sEVs, facilitating targeted delivery to GC tumors in a subcutaneous tumor-bearing nude mouse model. This approach effectively suppressed tumor proliferation and induced apoptosis both in vitro and in vivo, highlighting miR-13896 as a promising candidate for GC therapy.

## Conclusion

In conclusion, our study underscores the therapeutic potential of hucMSC-sEVs as effective and safe natural agents for inhibiting the malignant progression of GC. Our findings highlight the enrichment of various well-known anticancer miRNA molecules within hucMSC-sEVs, with particular emphasis on the novel miR-13896 identified in this study. Using targeted delivery through hucMSC-sEVs, miR-13896 effectively suppresses the growth and metastasis of GC cells by attenuating ATG2A-mediated autophagy. Furthermore, we have established a GC treatment platform that utilizes electroporation to engineer hucMSC-sEVs for enhanced delivery of miR-13896. This innovative approach not only validates miR-13896 as a promising therapeutic target for GC but also introduces engineered EVs as a novel strategy in GC treatment paradigms. In summary, our research contributes valuable information on the potential of miR-13896-loaded hucMSC-sEVs for precision therapy in GC. Moving forward, further investigation into the detailed mechanisms underlying miR-13896-mediated effects and clinical validation of engineered EVs, including its antitumor effects, will be crucial. These efforts are promising for advancing personalized treatment strategies and improving outcomes for patients with GC.

## Ethical Approval

All studies were approved by the Ethics and Experimental Animal Ethics Committee of Jiangsu University (IRB approval protocol number: 2020161). These studies strictly adhered to the principles outlined in the Declaration of Helsinki.

## Data Availability

The data used to support the findings of this work are available from the corresponding authors upon request.
