# Supplementary material for: Highly Efficient Delivery of Novel MiR-13896 by Human Umbilical Cord Mesenchymal Stem Cell-Derived Small Extracellular Vesicles Inhibits Gastric Cancer Progression by Targeting ATG2A-Mediated Autophagy
Source: Biomater Res. 2024 Dec 18;28:0119. doi: 10.34133/bmr.0119 (PMC11654722; doi:10.34133/bmr.0119)
Supplement: Supplementary 1 — Materials and Methods Figs. S1 to S8 Tables S1 and S2 [file bmr.0119.f1.docx]

**RESEARCH ARTICLE**

**Highly efficient delivery of novel miR-13896 by HucMSC-sEVs inhibits gastric cancer progression by targeting ATG2A-mediated autophagy**

Peipei Wu^1,2†*^, Min Wang^3†^, Can Jin^†4^, Linli Li^5^, Yuting Tang^4^, Zhangfei Wang^1,2^, Xianwen Wang^3^,Wenrong Xu^4*^, and Hui Qian^4*^

^1^Department of Laboratory Medicine, The First Affiliated Hospital of USTC, Division of Life Sciences and Medicine, University of Science and Technology of China, Hefei Anhui, 230001, China. ^2^Core Unit of National Clinical Research Center for Laboratory Medicine, Hefei Anhui, 230001, China. ^3^School of Biomedical Engineering, Research and Engineering Center of Biomedical Materials, Anhui Medical University, Hefei, Anhui, 230032, China. ^4^Jiangsu Key Laboratory of Medical Science and Laboratory Medicine, Department of Clinical Laboratory, School of Medicine, Jiangsu University, Zhenjiang, Jiangsu, 212000, China. ^5^Department of Clinical Laboratory, Changzhou Second Hospital, Changzhou, Jiangsu, 213000, China.

*Address correspondence to: [peipeiwu@ustc.edu.cn](mailto:icls@ujs.edu.cn;) (P.W.); [icls@ujs.edu.cn](mailto:icls@ujs.edu.cn;) (W.X.); [lstmmmlst@163.com](mailto:lstmmmlst@163.com) (H.Q.)

†These authors contributed equally to this work.

**Supplementary materials and methods**

**Identification of hucMSCs**

To confirm the multipotency of passage 3 hucMSCs, cells were seeded in 6-well plates at a density of 2×10⁴ cells/cm² and induced to differentiate into adipocytes and osteocytes using commercial differentiation kits (Cyagen Biosciences, HUXUC-90031, USA; Cyagen Biosciences, HUXUC-90021, USA). Adipogenic differentiation was assessed using Oil Red O staining, and osteogenic differentiation was evaluated with Alizarin Red S staining. HucMSC surface markers were detected according to the OriCell® mesenchymal stem cell (human) Surface Marker test kit Operating instructions (Cyagen Biosciences, HUXMX-09011, USA). The expression of surface markers on hucMSCs was determined by flow cytometry. The P3 hucMSCs were incubated with antibodies against CD11b, CD14, CD45, CD29, CD73, and CD105, or with isotype controls. After incubation with primary and secondary antibodies, cells were analyzed to verify the presence of these markers, indicative of the identity of hucMSC.

**Identification of the concentration and surface marks of hucMSC-sEVs**

The particle size, concentration, and zeta potential of the hucMSC-sEVs were measured using the Nanoparticle Tracking Analyzer (NTA; Germany, Particle Metrix, 220-Twin). Briefly, 1 μL of hucMSC-sEVs was diluted with PBS in an appropriate proportion and the concentration of hucMSC-sEVs was adjusted to ~1.0×10^8^–2.5×10^9^ cells/mL according to the detection concentration range of the instrument. A 5 mL disposable sterile syringe was used to inject the diluted hucMSC-sEVs into the sample detection chamber. The instrument performed a video capture, particle statistics, and an analysis of 11 sites on hucMSC-sEVs in stable Brownian motion according to the internal processing software. Western blotting was used to detect the expression of characteristic proteins on the surface and inside of hucMSC-sEVs. Positive markers of hucMSC-sEVs, such as Alix, TSG101, CD63, CD81, and negative control Calnexin, were determined by western blotting. The primary antibodies and their dilution ratios were as follows. Alix (1:500), TSG101 (1:500), CD63 (1:500), CD81 (1:500), Calnexin (1:500).

**Cellular uptake analysis**

A 1 mL-volume of hucMSC-sEVs was incubated with 5 μL of the membrane dye DIL (Termo, USA, D3911) for 30 min at 37 °C. The stained hucMSC sEVs were then transferred to 100 kDa MWCO ultrafltration centrifugal tubes (Millipore, USA). To remove unbound DIL, the samples were washed three times with PBS and centrifuged at 1500 x*g* for 20 min. DIL-labeled hucMSC-sEVs with the same particle number were administered to the MKN-45 and HGC-27 cells. Laser scanning confocal microscopy was applied to identify internalization of the DIL-labeled hucMSC-sEVs by MKN-45 and HGC-27 cells at different time points.

**Colony formation assay**

GC cells were harvested and seeded in 6-well plates and transfected with different treatment reagents for 48 h. A total of 2.5×10^3^ MKN-45 cells were seeded in 3.5 cm^2^ cell culture plates, and the complete fresh cell medium was replenished every three days. After approximately 14 days of culture, cells were washed with PBS three times and then 4% paraformaldehyde was added to fix the cells for 30 min at room temperature. After washing with PBS three times, crystal violet was added to stain the cells for 10 min at room temperature. The unbound dye was washed with PBS and the cells were dried, and the size and number of cell colonies were observed.

**Cell proliferation assay**

GC cells (5×10^3^ cells/well) were seeded in 96-well plates. After cells had adhered for 12–24 h, different concentrations of hucMSC-sEVs or miR-13896 mimic/inhibitor were added to each well for processing at different times. The Cell Counting Kit-8 (CCK-8) assay was performed to evaluate cell proliferation activity according to the manufacturer’s procedures. A volume of 100 µL of medium containing 10% CCK-8 reagent (Vazyme, Nanjing, China) was added to each well and incubated in the dark for 2 h at 37°C. The absorbance values of each well at 450 nm were measured by the Cytation 5 automatic microplate reader (BioTek, USA).

**Spheroid formation assay**

GC cells were seeded in 6‐well plates at a low density of 5000 cells per well and grown in serum‐free medium mix consisting of 200 mL DMEM/F‐12 or 200 mL RPMI 1640 basal media, 20 ng/mL EGF, 10 ng/mL bFGF, and 2% B27 (all from Sigma and Gibco). The number of spheroids per well was counted after 7 days under light microscopy at 40× magnification. The experiments were repeated at least three times.

**Flow cytometry for the detection of cell apoptosis**

GC cells were collected from different treatment groups. The cells were washed with precooled PBS twice and then resuspended in 1×10^7^ cells/mL in 1× cell staining binding buffer; 100 µL cell suspension was added to the flow tube, 5 µL FITC Annexin V was added followed by 10 µL PI. The cells were mixed in a gentle vortex and then incubated at room temperature for 15 min away from light; Finally, 400 µL Annexin V binding buffer was added to the flow tube and apoptosis was analyzed by flow cytometry after mixing.

**Lentiviral transfection**

The autophagy dual-labeled adenovirus (RFP-GFP-LC3) was obtained from GeneChem Biotechnology (Shanghai, China). MKN-45 cells were plated in 12-well plates at a density of 8×10⁴cells per well and incubated for 24 hours. Subsequently, an appropriate multiplicity of infection (MOI) of the adenoviral particles was added to the medium, followed by another incubation period of 24 hours. Stable transfected clones were selected by culturing in a suitable concentration of puromycin for two weeks.

**Western blotting**

Protein extraction from cells and sEVs was performed using RIPA buffer supplemented with protease inhibitors (Pierce, USA). Protein concentrations were quantified using a BCA protein assay kit. Equal amounts of protein were subjected to 12% SDS-PAGE, transferred to PVDF membranes (Millipore, USA), and blocked with 5% nonfat milk. Subsequently, the membranes were incubated with primary antibodies at 4°C overnight. The membranes were washed with 1× TBST and then incubated with an HRP-conjugated goat anti-rabbit/mouse IgG secondary antibody (USA, Invitrogen, 31460/31430) for 1 h at room temperature. Following washes, membranes were incubated with HRP-conjugated secondary antibodies (Invitrogen, USA) for 1 h at room temperature, and bands were visualized. The primary antibodies used in this study were: Bax (CST, USA, 2772S), Bcl2 (CST, USA, 15071S), PCNA (CST, USA, 4711S), CyclinD3 (CST, USA, 2936S), Vimentin (CST, USA, 5741S), Snail (CST, USA, 3895S), Slug (CST, USA, 9585P), E-cadherin (CST, USA, 3195S), N-cadherin (CST, USA, 13116P), p-AKT (CST, USA, 4060S), t-AKT (SAB, USA, 21501), ATG2A (Invitrogen, USA, MA5-31639), LC3B (CST, USA, 2775S), Beclin1 (CST, USA, 3495S), P62 (CST, USA, 39749S), CD63 (Abcam, USA, ab59479), CD81(Proteintech, USA, 18250-1-AP), Alix (CST, USA, 2171S), TSG101 (Abcam, USA, ab30871), Calnexin (CST, USA, 4872S), and β-actin (Bioworld, USA, AP0060).

**Figures and Tables**


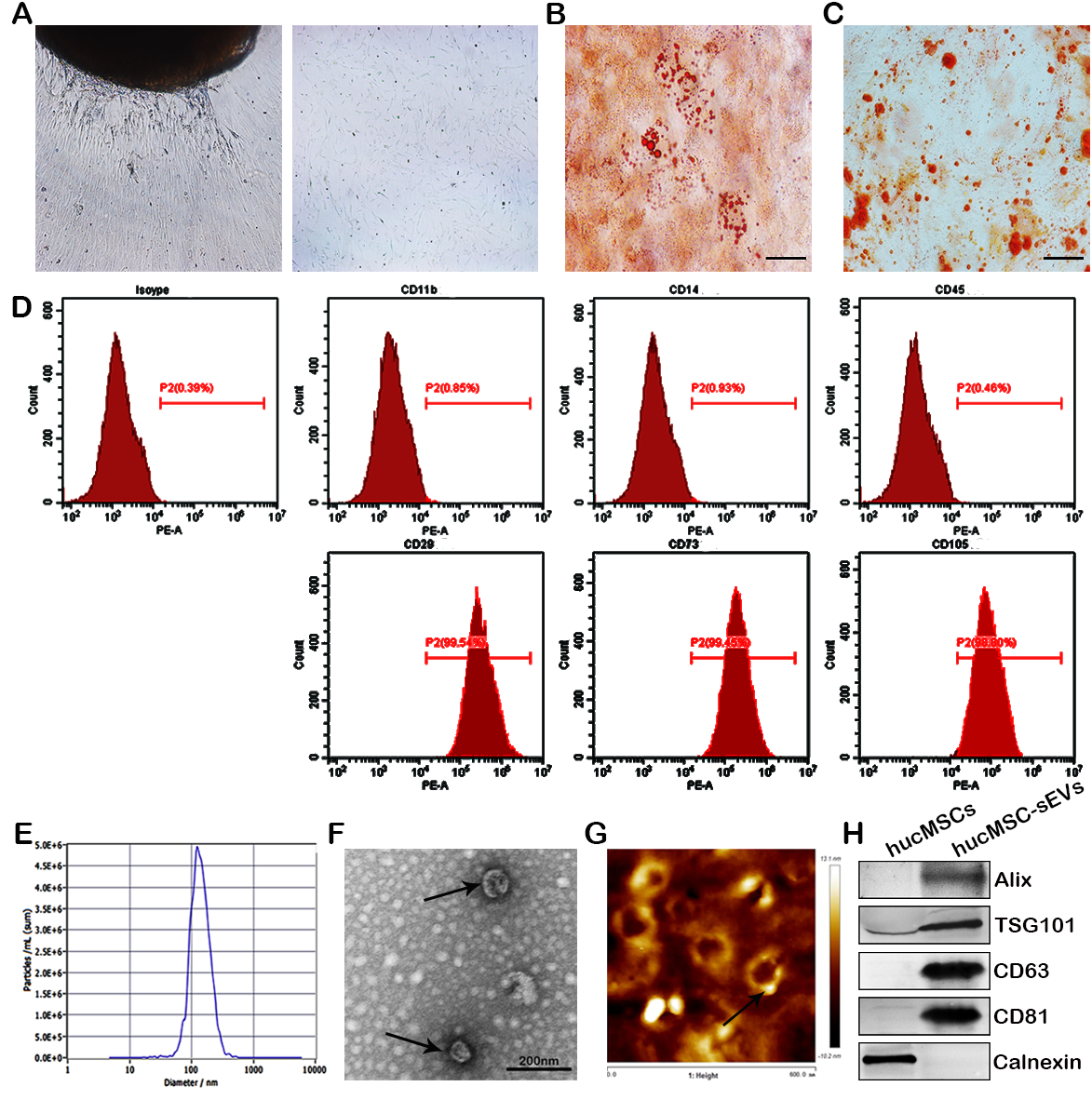


**Fig. S1.** Isolation and characterization of hucMSCs and hucMSC-sEVs. (**A**) The morphology of primary (Magnification, 40 x) and passage 3 hucMSCs (Magnification, 100x) were observed under a light microscope. (**B**&**C**) Representative images of adipocytes (100x) and osteocytes (100x) differentiation of p3 hucMSCs cultured in the adipogenic and osteogenic differentiation media. The cells were analyzed using cytochemical staining with Oil red O and Alizarin Red, respectively. (**D**) ZetaView was used to examined the size distribution of the hucMSC-sEVs. (**E**) Transmission electron microscopic images of hucMSC-sEVs (Scale bar=200 nm). (**F**) Atomic force microscope of hucMSC-sEVs (Scale bar=600 nm). (**G**) The positive (CD9, CD63, CD81, Alix, and TSG101) and negative (Calnexin) expression of markers in hucMSC-sEVs was detected by Western blotting.


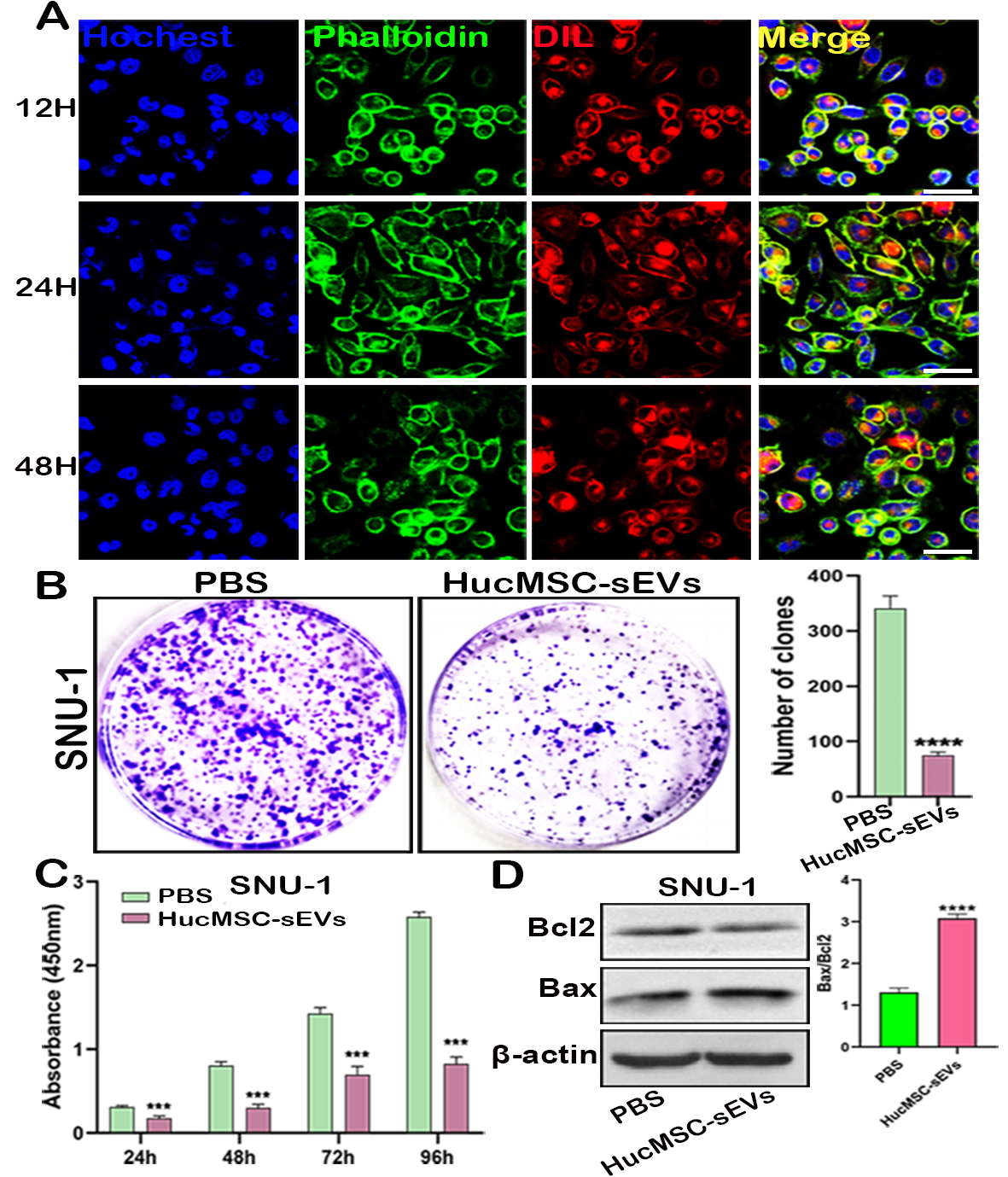


**Fig. S2.** HucMSC-sEVs inhibit GC cell proliferation and promote apoptosis. (A) Confocal microscopy to detect the uptake of DIL-labeled hucMSC-sEVs by the GC cell lines SNU-1 cells (Phalloidin, green fluorescence; DIL, Red fluorescence). (B) The clone formation assay detected cell proliferation of GC cell lines SNU-1 cells after treatment with hucMSC-sEVs for 48 h. (C) CCK-8 assay detected the proliferation of GC cell lines SNU-1 after treatment with hucMSC-sEVs at different time points. (D) Western blotting to detect apoptosis of GC cell lines SNU-1 cells after treatment with hucMSC-sEVs for 48 h.


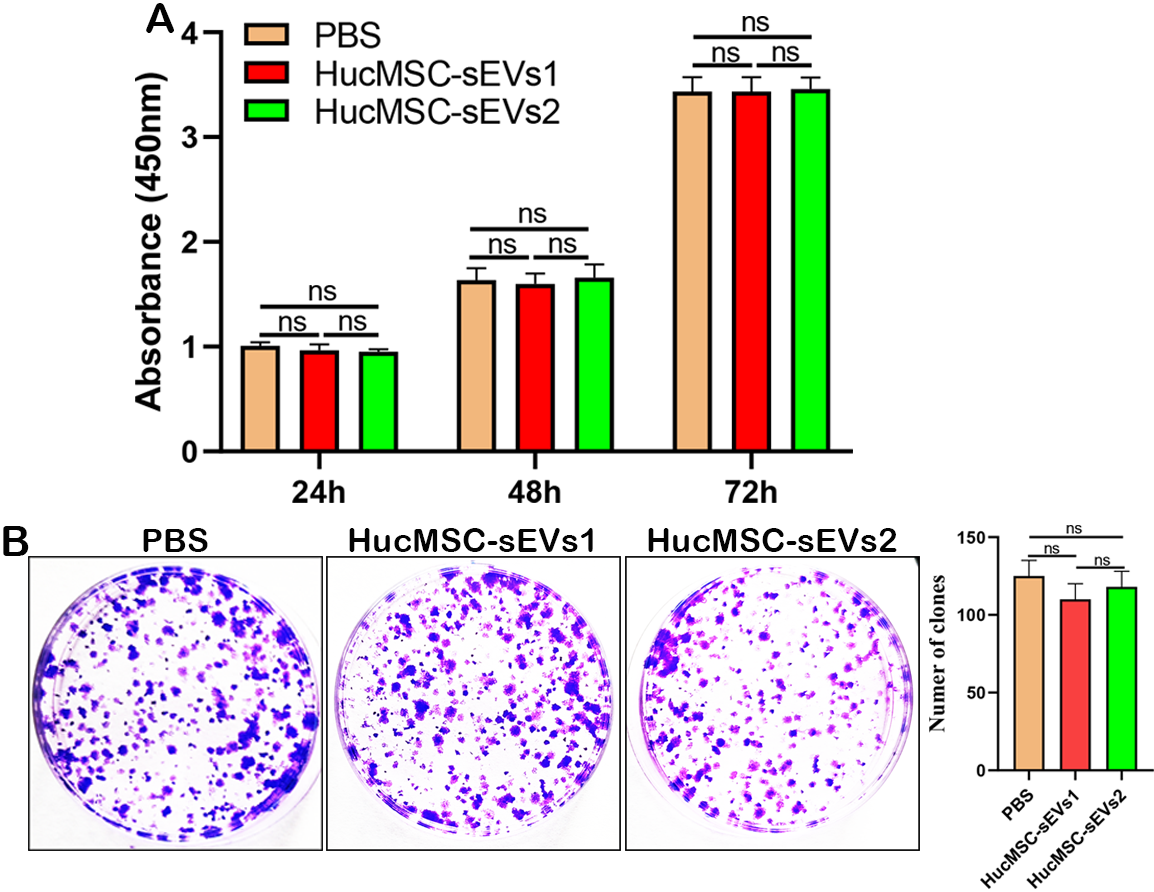


**Fig. S3.** Safety evaluation of hucMSC-sEVs intervention GES-1. (A) CCK-8 assay detected the proliferation of GC cell lines SNU-1 after treatment with different concentrations of hucMSC-sEVs (hucMSC-sEVs1, 1x10^8^ Particle; hucMSC-sEVs2, 1x10^9^ Particle) at different time points. (B) The clone formation assay detected cell proliferation of GC cell lines SNU-1 cells after treatment with hucMSC-sEVs different concentrations of hucMSC-sEVs (hucMSC-sEVs1, 1x10^8^ Particle; hucMSC-sEVs2, 1x10^9^ Particle) for 48 h.


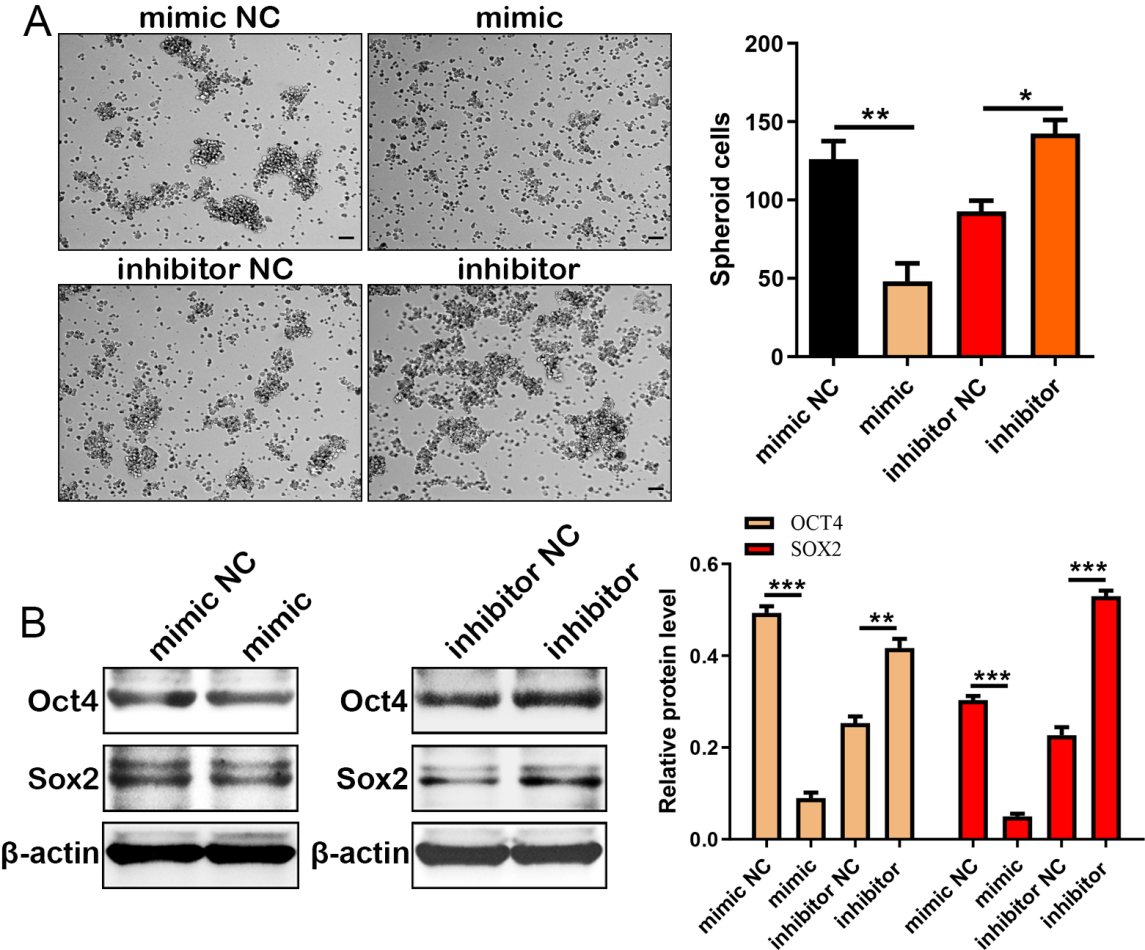


**Fig. S4.** miR-13896 inhibited stem cell gene expression and self-renewal of GC cells. **(A**) Western blotting for detecting the expression of transcription factors Oct4 and Sox2 in MKN-45 cells transfected with miR-13896 mimic and inhibotor. (**B**) Representative images show decreased numbers and volume of mammospheres of MKN-45 after treating with miR-13896 mimic and inhibotor.


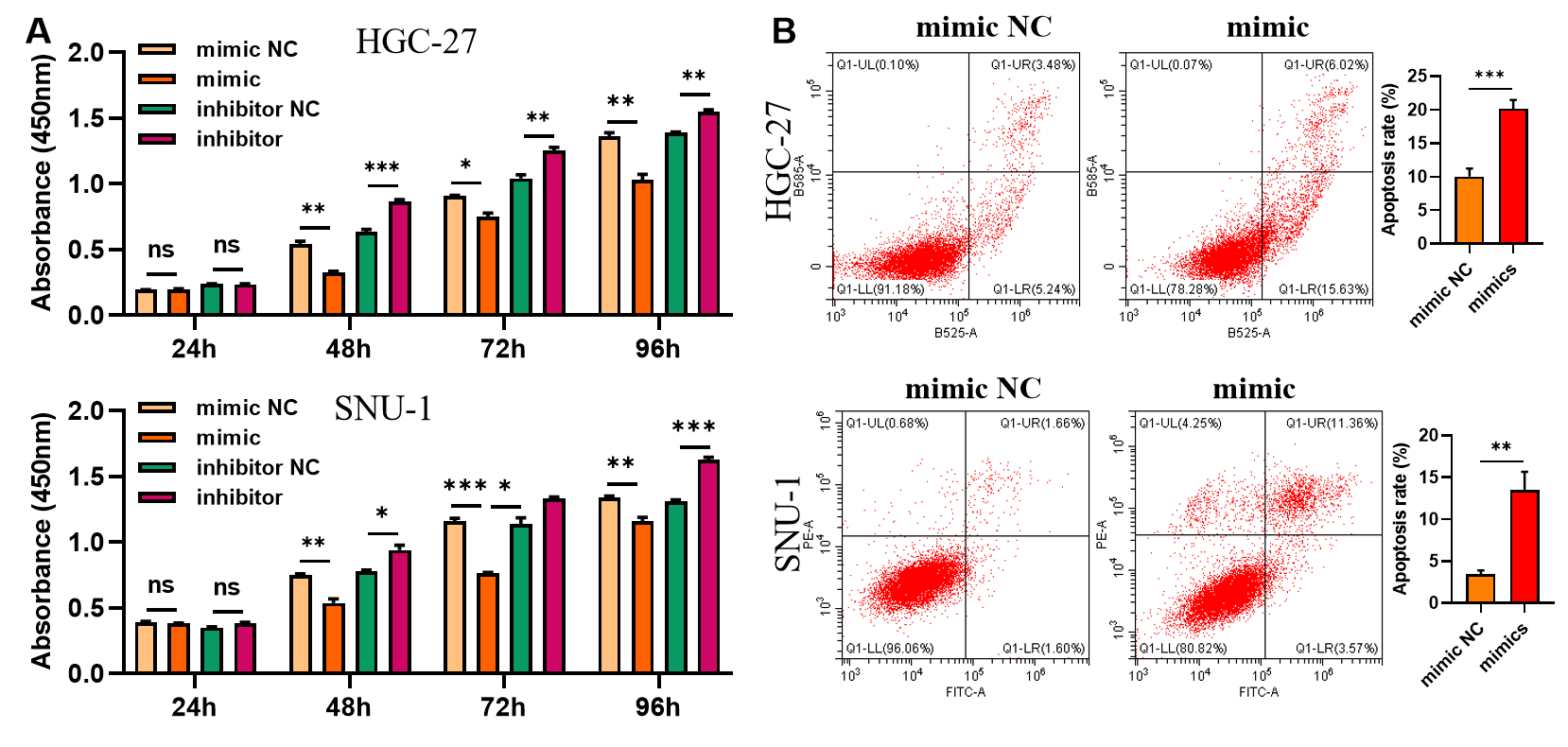


**Fig. S5.** miR-13896 inhibits the proliferation and metastasis of HGC-27 and SNU-1 cells. (A) CCK-8 assay detected the proliferation of GC cell lines HGC-27 and SNU-1 after treatment with miR-13896 engineered hucMSC-sEVs at different time points. (B) Flow cytometry was used to detect the apoptosis of HGC-27 and SNU-1 cells transfected with 5 nmoL concentration of miR-13896 mimic for 48 h.


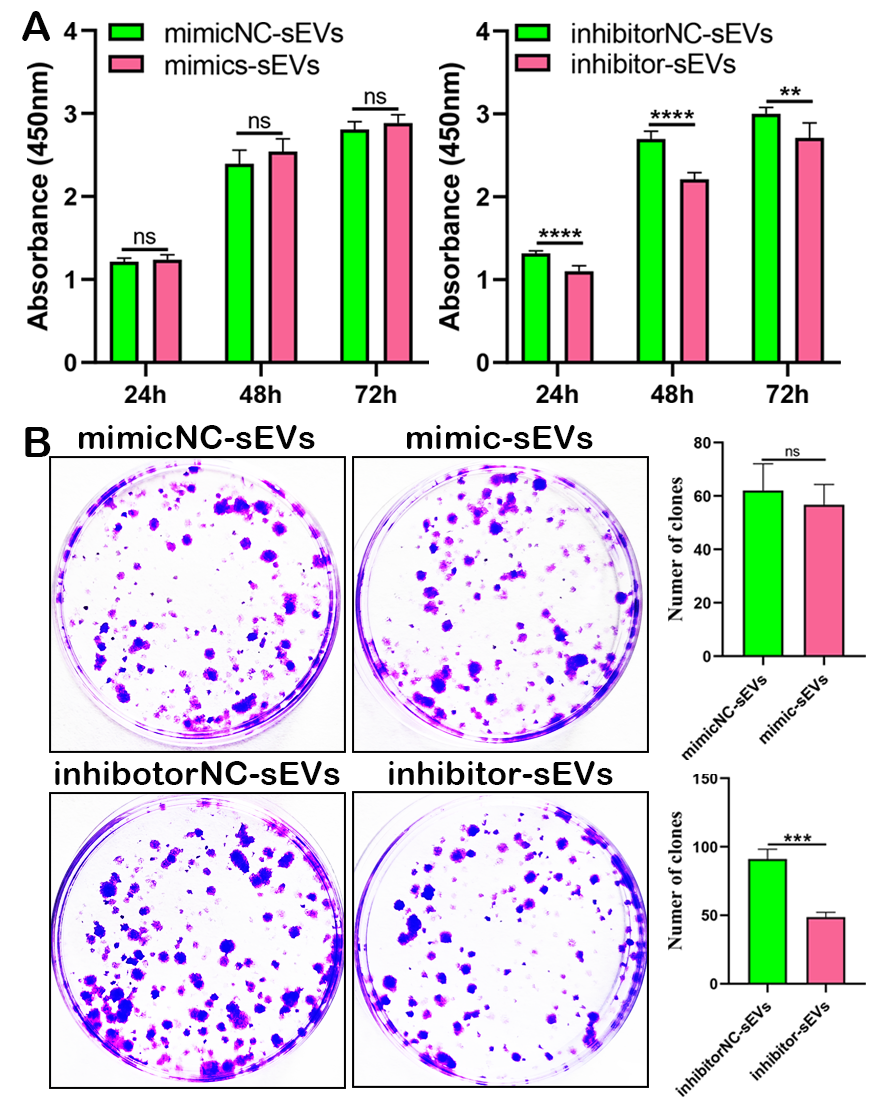


**Fig. S6.** Safety evaluation of miR-13896 engineered hucMSC-sEVs intervention GES-1. (A) CCK-8 assay detected the proliferation of GC cell lines SNU-1 after treatment with miR-13896 engineered hucMSC-sEVs at different time points. (B) The clone formation assay detected cell proliferation of GC cell lines SNU-1 cells after treatment with miR-13896 engineered hucMSC-sEVs for 48 h.


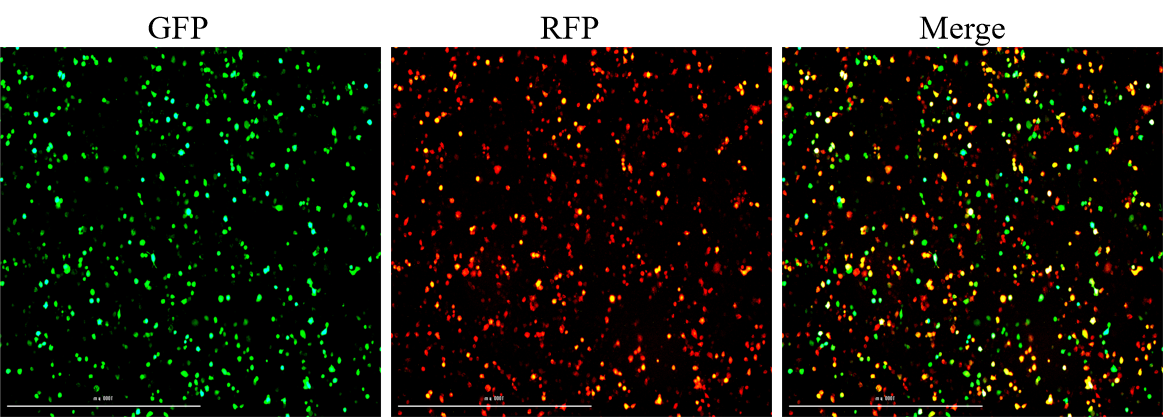


**Fig. S7.** The multimode reader was used to detect the fluorescence efficiency of GFP-RFP-LC3 double-marking autophagy adenovirus transfected into MKN-45 cells for 72 h.


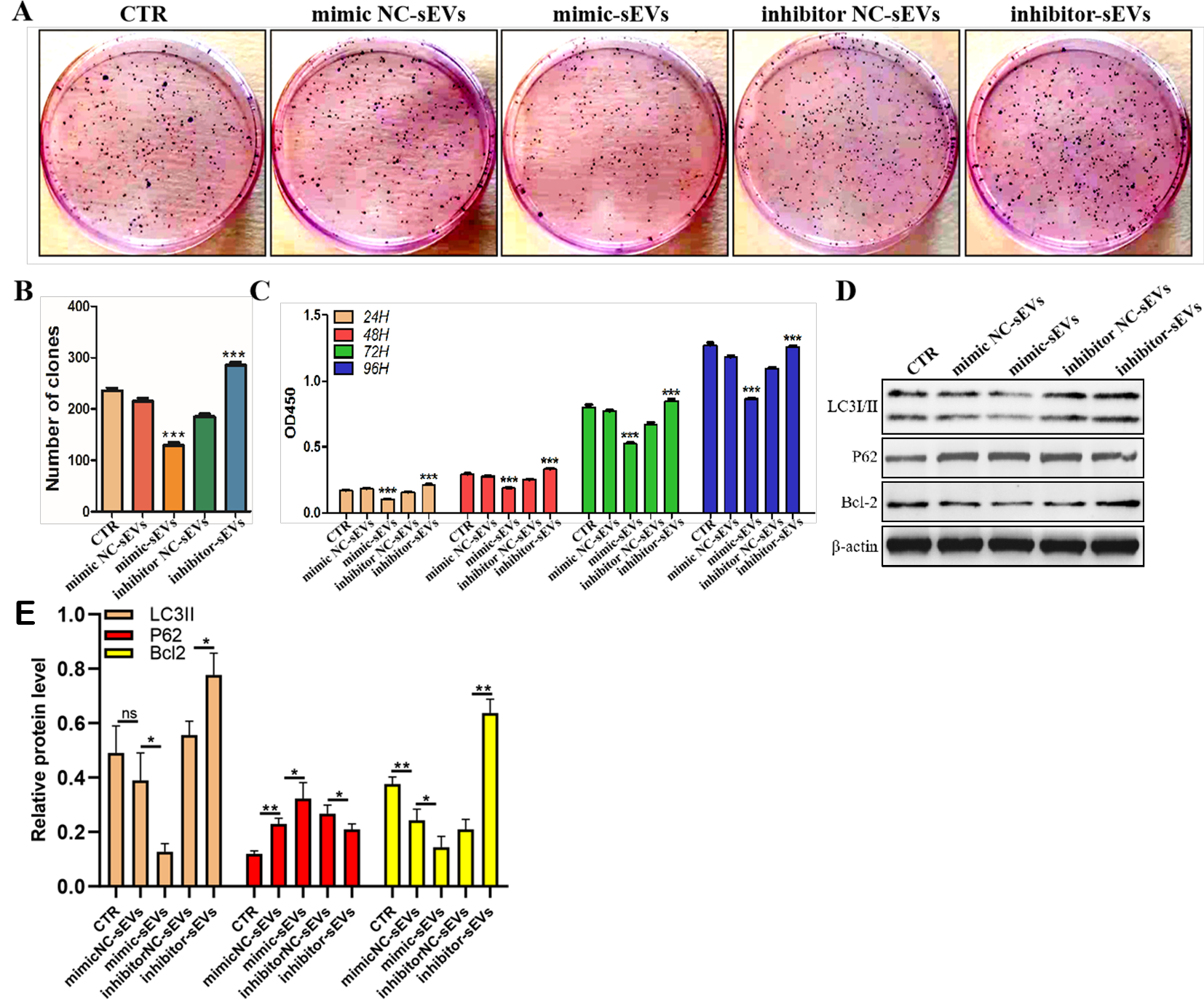


**Fig. S8.** miR-13896 engineered hucMSC-sEVs enhances its ability to inhibit GC cell proliferation and autophagy. **(A**) The effect of miR-13896 engineered hucMSC-sEVs on the formation of GC cell MKN-45 colony detected by clonogenesis assay. (**B**) Clone statistical results of Figure S8A. (**C**) CCK-8 detected the effect of miR-13896 engineered hucMSC-sEVs on the proliferation activity of GC cell MKN-45. (**D**) Western blotting analysis of the autophagy effect of miR-13896 engineered hucMSC-sEVs on GC cells MKN-45.（**E**）Densitometric analysis of protein bands in Figure S8D.

**Table 1.** Sequences of real-time PCR primers.

| Gene names | Primer | Sequences  (5′-3 ′) | Fragment  Size | Annealing temperature |
| --- | --- | --- | --- | --- |
| miRNA13896 | Forward | CCTTGGGATGGTCTGGACTG | 70 bp | 60℃ |
|  | Reverse | TATGGTTGTTCACGACTCCTTCAC |  |  |
| U6 snRNA | Forward | CGCTTCGGCAGCACATATAC | 87 bp | 60℃ |
|  | Reverse | TTCACGAATTTGCGTGTCATC |  |  |
| Bax | Forward | CACCAGCTCTGAGCAGATCAT | 214 bp | 61℃ |
|  | Reverse | GATCAGTTCCGGCACCTTG |  |  |
| Bcl2 | Forward | GGATCCAGGATAACGGAGGC | 150 bp | 60℃ |
|  | Revrse | CCAGATAGGCACCCAGGGT |  |  |
| ATG2A | Forward | GCTCAGGGTACATGGAGCTG | 167 bp | 60℃ |
|  | Revrse | CTCGTGGTCTGTAAGGCTCAC |  |  |
| β-actin | Forward | GACCTGTACGCCAACACAGT | 129 bp | 59℃ |
|  | Reverse | CTCAGGAGGAGCAATGATCT |  |  |

**Table 2**. Small interfering RNA sequence.

| human ATG2A | sense（5'-3'） | antisense（5'-3'） |
| --- | --- | --- |
| ATG2A-homo-168 | GCUACUUGCUGCACCACUATT | UAGUGGUGCAGCAAGUAGCTT |
| ATG2A-homo-479 | GCCUCAUGCAUGACCACAATT | UUGUGGUCAUGCAUGAGGCTT |
| ATG2A-homo-1485 | CCGAGUUUGAUGCCACCAATT | UUGGUGGCAUCAAACUCGGTT |
